# Single-cell profiling of human subventricular zone progenitors identifies SFRP1 as a target to re-activate progenitors

**DOI:** 10.1038/s41467-022-28626-9

**Published:** 2022-02-24

**Authors:** Vanessa Donega, Astrid T. van der Geest, Jacqueline A. Sluijs, Roland E. van Dijk, Chi Chiu Wang, Onur Basak, R. Jeroen Pasterkamp, Elly M. Hol

**Affiliations:** 1 Department of Translational Neuroscience, UMC Utrecht Brain Center, University Medical Center Utrecht, Utrecht University, 3584 CG Utrecht, The Netherlands; 2 Department of Obstetrics and Gynecology, Li Ka Shing Institute of Health Sciences, School of Biomedical Sciences, and Chinese University of Hong Kong -Sichuan University Joint Laboratory in Reproductive Medicine, The Chinese University of Hong Kong, Shatin, NT Hong Kong; 3 Institute of Biochemistry, Charite-University Medicine Berlin, Charitéplatz 1, Berlin, Germany

**Keywords:** Adult neurogenesis, Neural stem cells, Ageing, Cell signalling

## Abstract

Following the decline of neurogenesis at birth, progenitors of the subventricular zone (SVZ) remain mostly in a quiescent state in the adult human brain. The mechanisms that regulate this quiescent state are still unclear. Here, we isolate CD271^+^ progenitors from the aged human SVZ for single-cell RNA sequencing analysis. Our transcriptome data reveal the identity of progenitors of the aged human SVZ as late oligodendrocyte progenitor cells. We identify the Wnt pathway antagonist SFRP1 as a possible signal that promotes quiescence of progenitors from the aged human SVZ. Administration of WAY-316606, a small molecule that inhibits SFRP1 function, stimulates activation of neural stem cells both in vitro and in vivo under homeostatic conditions. Our data unravel a possible mechanism through which progenitors of the adult human SVZ are maintained in a quiescent state and a potential target for stimulating progenitors to re-activate.

## Abstract (web summary)

The decline in neurogenesis following birth is accompanied with a quiescent state characteristic of neural progenitors of the adult brain. Here, the authors identify the Wnt pathway antagonist SFRP1 as a potential signal that promotes quiescence and show that its inhibition stimulates stem cell activation.

## Introduction

In most mammals, neurogenesis in the dentate gyrus (DG) and subventricular zone (SVZ) continues during adulthood^1^. In rodents and non-human primates, new neurons generated in the SVZ migrate to the olfactory bulb. In humans, on the other hand, the addition of new neurons to the olfactory bulb is likely negligible^1–5^ and new neurons produced in the SVZ migrate to the neighboring striatum^2^. Growing evidence suggests that the decline in neurogenesis observed during aging in mammals is due to increased quiescence of neural stem cells (NSCs) and progenitors^6–8^ (hereafter progenitors refers to both NSCs and progenitors).

Studies in rodents have shown that adult NSCs arise from a population of quiescent radial glial cells that accumulate embryonically^9,10^. Rather than being a static non-proliferating pool of cells, studies in rodents have demonstrated that they are a very dynamic population of cells that transit between proliferative and quiescent states^11–13^. With aging progenitors become less plastic and remain mainly quiescent, which prevents depletion of the progenitor pool^7^. The mechanisms that regulate quiescence of progenitors are just beginning to be unraveled^6–8,14–23^. With age, the germinal niches become less neurogenic due to increased inflammatory signals and Wnt pathway antagonists, and decreased activity of the Wnt pathway^6,7,12,24–26^. Despite the decrease in neurogenic function of the aged SVZ, adult progenitors are permissive to pharmacological or genetic approaches that stimulate their neurogenic potential^7,27,28^. Furthermore, progenitors were shown to exit quiescence and re-enter the cell-cycle following ischemic injury in adult rodents^12^. Quiescent NSCs of the SVZ could be a potential source of stem cells for repair. However, the transcriptional programs that regulate quiescence of progenitors of the human brain are still unclear.

We have previously identified NGFR (i.e. CD271) as a marker expressed by progenitors of the aged human SVZ^29–31^. We showed that these cells form neurospheres and can differentiate into immature neurons and glia cells in vitro^30^. The present study assesses the molecular identity of NGFR-positive progenitors from the SVZ of the aged human brain at single-cell level and investigates a mechanism through which human progenitors could be maintained in a quiescent state. We identify the secreted frizzled-related protein-1 (SFRP1), an inhibitor of the Wnt signaling pathway, to be among genes whose expression changes over time. We demonstrate that inhibition of SFRP1 with a small molecule stimulates proliferation in vitro, in human iPSC-derived NSCs, and in vivo in early postnatal mice. Altogether, our work proposes a mechanism that maintains quiescence of progenitors of the human SVZ, which opens up future possibilities to stimulate NSCs of the human brain to promote repair.

## Results

### Characterization of the human SVZ at single-cell level

**Fig. 1:**
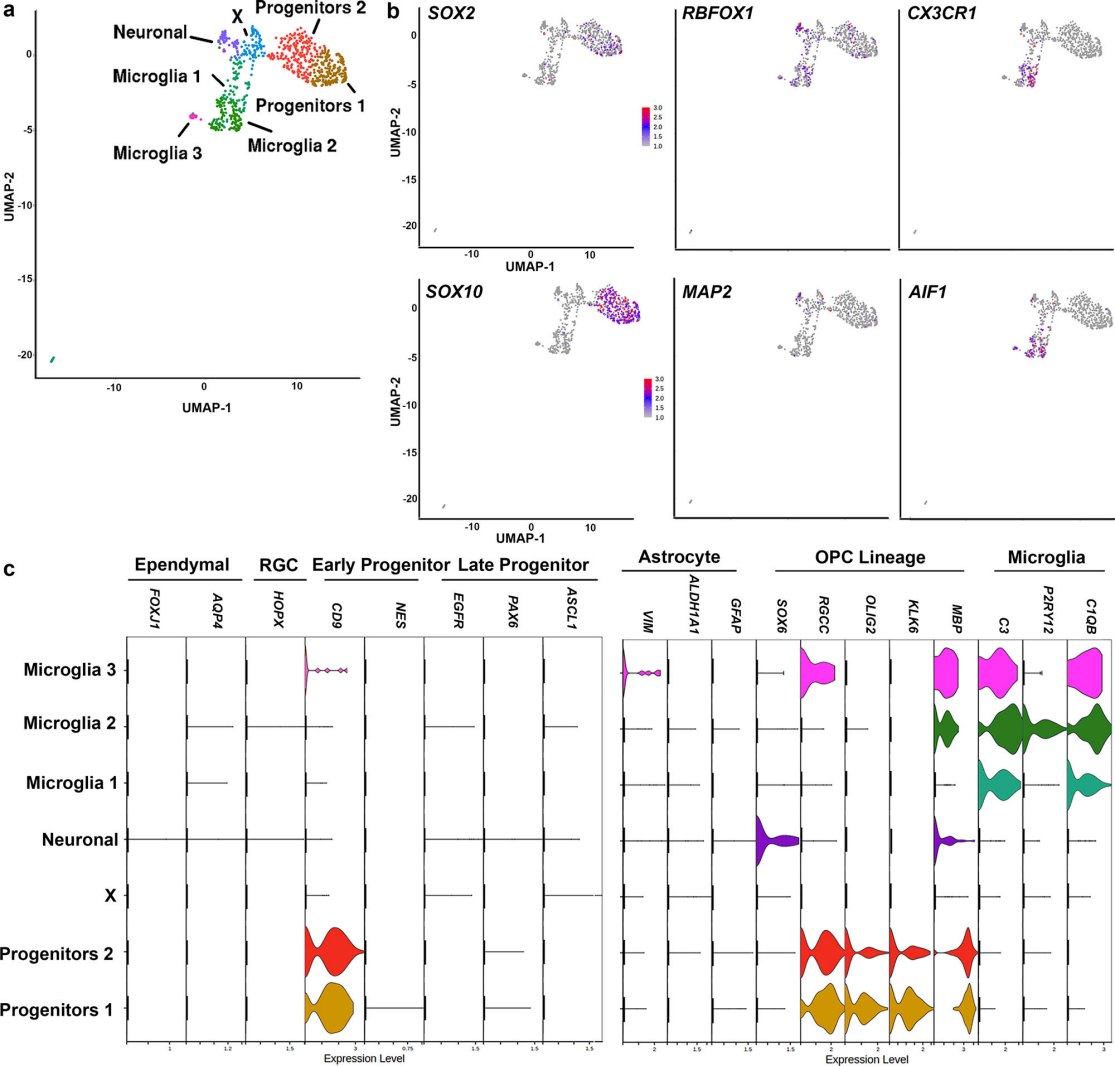
Characterization of the aged human SVZ at the transcriptome level. **a** UMAP plot identifies seven clusters of cells, corresponding to two progenitor clusters, one neuronal, and three microglia clusters. **b** Feature plots showing the expression of canonical markers for the clusters: Progenitor 1 and 2 *(SOX2; SOX10)*, Neuronal (*RBFOX1; MAP2*), and Microglia 1, 2, and 3 *(CX3CR1; AIF1*). **c** Feature plots showing the absence or presence of markers for ependymal, radial glial cell (RGC), early progenitor, late progenitor, astrocytes, oligodendrocyte progenitor cell (OPC) lineage and microglia. (*n* = 3 donors; 2 females, aged 95 (504 cells) and 96 years (118 cells); 1 male, aged 72 years (116 cells)).

We have recently confirmed the progenitor identity of NGFR^+^ (i.e. CD271) cells^31^ from the human SVZ by assessing their transcriptome and proteome signature. To further characterize the dorsal SVZ of the aged human brain we performed single-cell RNA sequencing, on the freshly isolated dorsal SVZ from donors without known neurological or psychiatric disease (*n* = 3, mean age = 88; 2 females, aged 95 (504 cells, mean UMI per cell 3935, mean gene number per cell 1352) and 96 years (118 cells, mean UMI per cell 4181, mean gene number per cell 873); 1 male, aged 72 years (116 cells, mean UMI per cell 3991, mean gene number per cell 779)) (Supplementary Data 1). Progenitors, astrocytes, and microglia were isolated by fluorescently labeling the different populations for CD271 (progenitors)^31^, GLT-1 (astrocytes), and CD11b (microglia), followed by FACS (Supplementary Fig. 1a, b). We also sorted the negative fraction. We obtained the profile of 1074 cells from the SVZ of the aged human brain. After QC-analysis, 738 cells remained for further analysis (Supplementary Fig. 1c–h). We performed unbiased cluster analysis using the Louvain algorithm and the Uniform Manifold Approximation and Projection (UMAP)^32,33^ identifying seven clusters (Fig. 1a and Supplementary Fig. 2a). Cell types clustered based on biological cell type, rather than donor or technical artefacts (Supplementary Fig. 1d, e). We identified three microglia clusters, viz. Microglia 1, Microglia 2, and Microglia 3 as they expressed canonical microglia markers (e.g. *CX3CR1* and *AIF1*) (Fig. 1a, b). These three clusters contained cells that were CD11b^+^. We identified two clusters as progenitor clusters (i.e. Progenitors 1 and Progenitors 2), which expressed markers for progenitors (e.g. *SOX2* and *SOX10*), and lacked expression of markers for ependymal cells (*FOXJ1* and *AQP4*), radial glial cells (*HOPX*), or astrocytes (*VIM*, *GFAP*, and *ALDH1A1*) (Fig. 1c). These two clusters contained the cells that were sorted based on CD271 expression and some cells from the negative fraction (Supplementary Fig. 1e–h). They expressed the marker for early progenitor/astrocyte *CD9*^12^, but did not express markers for activated progenitors *NES* and *EGFR*^22^ or markers for late neuronal progenitors (e.g*. PAX6* and *ASCL1*). Both clusters also expressed markers for the oligodendrocyte lineage including the oligodendrocyte progenitor cell (OPC) markers *SOX10* and *RGCC*^34^. The cluster Neuronal was negative for all the above markers, and instead, expressed *SOX6* and neuronal markers (e.g*. MAP2, RBFOX1*, *NRXN1,* and *CTNNA2*) (Fig. 1b, c and Supplementary Fig. 2). *SOX6* is a transcription factor expressed in early OPCs, but has also been associated with the development of interneurons^35–37^. The final cluster that we identified only expressed *LYZ* and *SPINT2* as cluster marker genes (Supplementary Fig. 2 and Supplementary Data 2). These two clusters only contained CD271^-^CD11b^-^GLT1^-^ sorted cells.

To further substantiate the identity of the two Progenitor and the Neuronal clusters we performed Gene ontology (GO) analysis on all highly expressed genes (adj *P*-value < 0.01) (Supplementary Data 2) within the clusters: Progenitors 1, Progenitors 2, and Neuronal. Cluster Progenitors 1 and 2 showed enrichment for GO terms related to central nervous system development, axonogenesis, and glial cell development (Supplementary Fig. 3). Cluster Neuronal showed enrichment for terms related to protein modification, cell adhesion, and glutamate receptor binding. These GO analyses corroborate the identities of the three clusters as progenitors and neuronal.

### Progenitors isolated from the aged human SVZ are OPCs

**Fig. 2:**
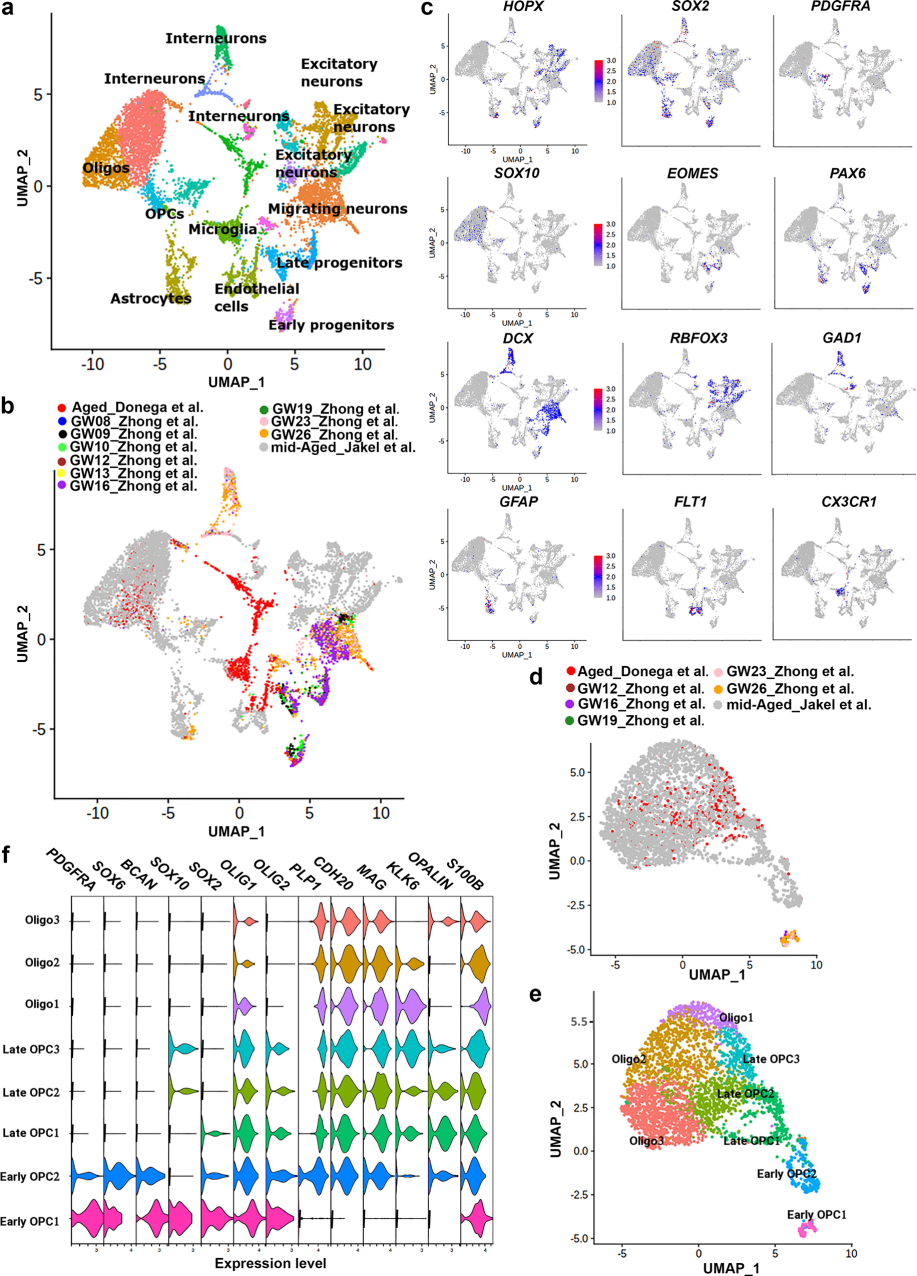
CD271^+^ cells correspond to late OPCs and oligodendrocytes. **a**, **b** UMAP projection of cell clusters shown by cell population (**a**) and origin of individual cells (**b**). **c** Feature plots for canonical markers of different cell populations. **d**, **e** UMAP projection of the origin of individual cells (**d**) and OPC lineage subclusters (**e**). **f** Violin plots of markers enriched in specific oligodendrocyte subpopulation. Gene expression level plotted as normalized counts. GW gestational weeks. (*n* = 3 donors; 2 females, aged 95 (504 cells) and 96 years (118 cells); 1 male, aged 72 years (116 cells)).

As the gene signature of aged human SVZ progenitors suggested an OPC identity we compared our data to the dataset from Zhong et al.^38^ and Jäkel et al.^39^. Zhong et al.^38^ isolated cells from the fetal human brain at different gestational stages and Jäkel et al.^39^ isolated white matter cells from healthy donors aged between 35 and 82 years (mean age 60 years). We used Seurat v3.2.2 to run an integrative analysis on the three datasets. This revealed several clusters including early progenitors, late progenitors, and migrating neurons (Fig. 2a–c). Moreover, we observed substantial mixing of cells from the different datasets, arguing against clustering due to batch effects. Neuronal lineage clustered together, and include mostly fetal cells and mid-aged cells from Jäkel et al^39^. Microglia from our dataset clustered with microglia from the two other studies. Cells from the OPC lineage from our dataset formed clusters with OPC lineage cells from Jäkel et al.^39^, and fetal OPCs (Fig. 2a, b and Supplementary Data 3). We next performed clustering analysis on cells from the OPC lineage only, which revealed seven subclusters (Fig. 2d, e). These subclusters corresponded to early OPCs (*PDGFRA* and *SOX6*), late OPCs (*SOX10* and *SOX2*), and oligodendrocytes (*KLK6* and *OPALIN*) (Fig. 2f). Our analysis showed that from the 395 progenitor cells that we analyzed, 138 cells corresponded to late OPCs and the remaining cells to oligodendrocytes (Supplementary Data 3). We performed SOX10 immunofluorescence staining on post-mortem human brain tissue, which showed that only a few SOX2 progenitors in the SVZ are SOX10 positive (Supplementary Fig. 4).

### Cell-cycle inhibitors increase in OPCs from the aged SVZ

**Fig. 3:**
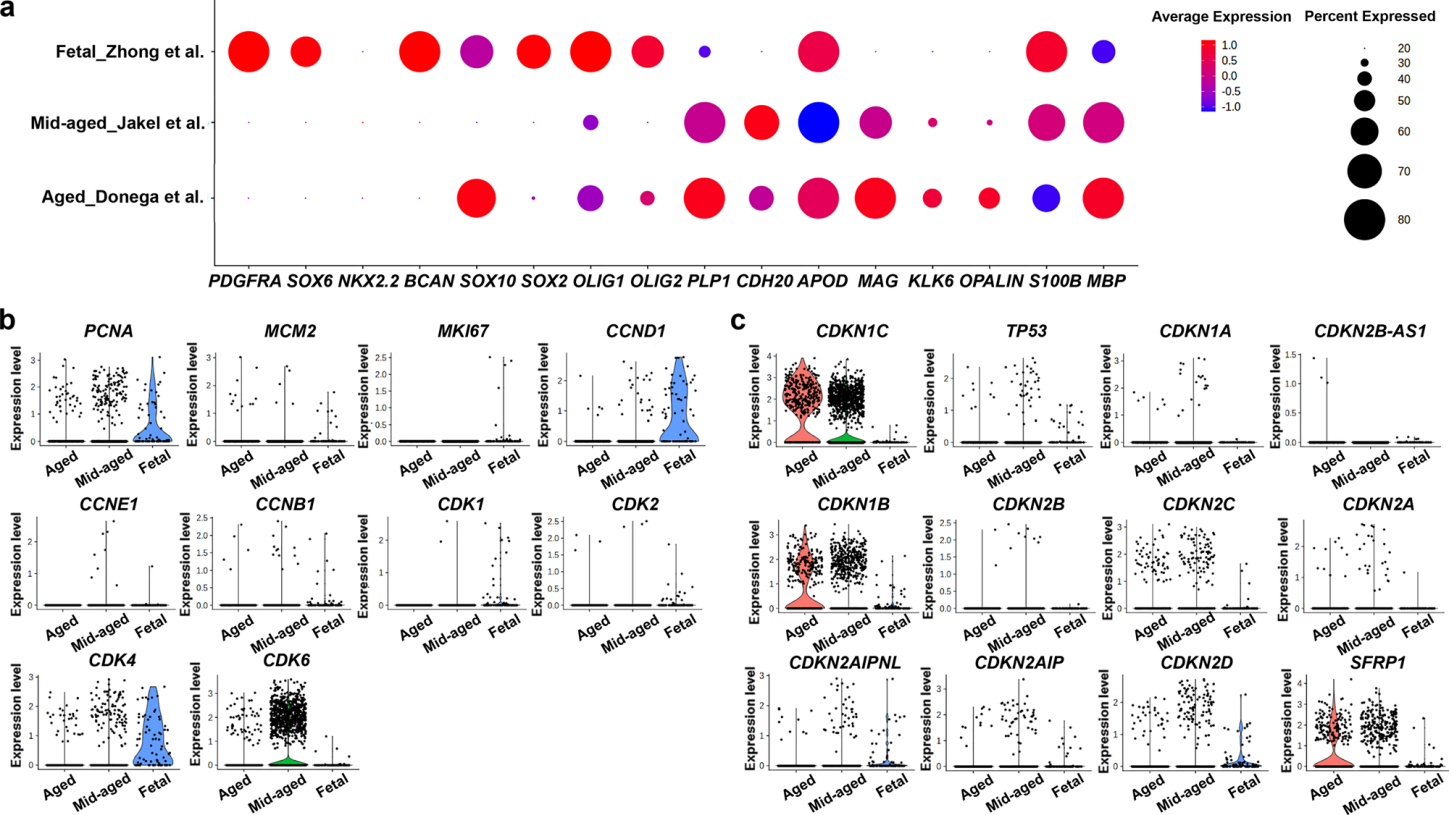
Increased expression of cell cycle inhibitors in OPCs from the aged human SVZ. **a** Dot plot showing the expression level (red = high, blue = low) and percentage of cells expressing markers of the oligodendroglial lineage. **b**, **c** Violin plots of markers for proliferation and cell cycle progression (**b**) and of markers for quiescence and cell cycle inhibition (**c**) in cells from the OPC lineage from Zhong et al.^39^, Jäkel et al.^42^, and present study (Donega et al.). (*n* = 3 donors; 2 females, aged 95 (504 cells) and 96 years (118 cells); 1 male, aged 72 years (116 cells)).

Analysis of the expression of a panel of markers for the oligodendroglial cell lineage further confirmed the OPC identity of the CD271^+^ progenitor cells (Fig. 3a). We next identified genes that were differentially expressed over time using Monocle3 v0.2.3.0 (Supplementary Data 4). One of the genes that was differentially expressed over time was *SFRP1*, which increased in expression with age (Supplementary Data 4). SFRP1 is an antagonist of the Wnt pathway, thereby inhibiting cell proliferation^40,41^. This is interesting as the mechanisms that regulate quiescence of progenitors from the human SVZ are unclear. Therefore, we compared the expression of several proliferation and cell cycle markers in fetal, mid-aged, and aged OPC lineage cells. As expected, markers for proliferation and cell cycle progression were mostly absent in the mid-aged and aged OPC lineage cells (Fig. 3b), while markers for quiescence and cell cycle arrest were highly expressed in mid-aged and aged OPC lineage cells, in particular *CDKN1B* (i.e. *P27*), *CDKN1C* (i.e. *P57*), and *SFRP1* (Fig. 3c).

### Cell-cycle inhibitors are expressed in the aged human SVZ

**Fig. 4:**
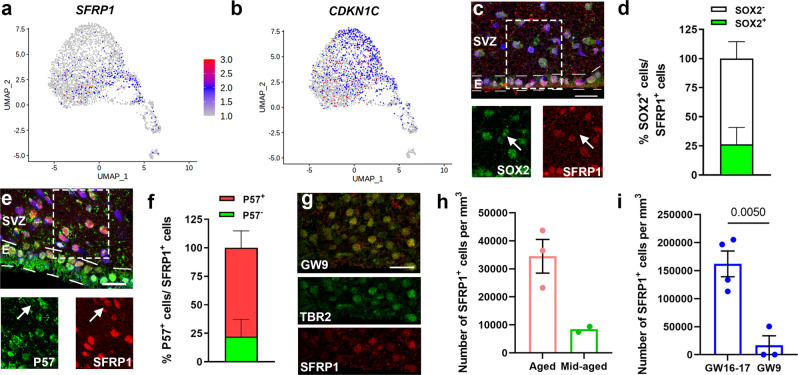
Cell cycle inhibitors are expressed in the aged human SVZ. **a**, **b** Feature plot showing the expression of *CDKN1C* (*P57*) (**a**) and *SFRP1* (**b**) (*n* = 3 donors; 2 females, aged 95 (504 cells) and 96 years (118 cells); 1 male, aged 72 years (116 cells)). **c** Representative image of SFRP1^+^ progenitors in the aged human SVZ. **d** Quantification of the percentage of SFRP1^+^ cells that express the stem cell marker SOX2 (*n* = 3 donors). **e** Representative image of SFRP1^+^ and P57^+^ cells in the aged human SVZ. **f** Quantification of the proportion of SFRP1^+^ cells that express P57 (*n* = 3 donors). **g** Representative images of SFRP1^+^ cells in the germinal region of the fetal human brain at GW9 co-expressing TBR2. **h** Quantification of SFRP1^+^ cells in the SVZ of aged (*n* = 3 donors; 2 females aged 92 and 99 years, 1 male aged 82 years) and mid-aged (*n* = 2 donors; 2 females aged 62 and 50 years) post-mortem brain tissue. **i** Quantification of SFRP1^+^ cells in the SVZ of GW9 (*n* = 3 donors; 2 females, 1 male) and GW16-17 (*n* = 4 donors; 2 females, 2 males) post-mortem brain tissue. Data represented as mean ± SEM when *n* > 2. Data represented as mean when *n* = 2. Two-tailed Unpaired Student’s *t* test. Hoechst was used as a nuclear counterstaining. E ependymal layer, GW gestational weeks. Scale bar = 20 µm. Source data are provided as a Source Data file.

P57 is a known marker for stem cell quiescence in rodents^9,23^ and SFRPs are a family of biphasic regulators of Wnt signaling expressed in the nucleus or cytoplasm of the cell^40–42^. *SFRP1* is mainly expressed in late OPCs (Fig. 4a) and is the only member of the SFRP family that is expressed in aged OPCs (Supplementary Fig. 5a). In contrast, *P57* is expressed in both late OPCs as well as oligodendrocytes (Fig. 4b). To characterize the expression pattern of both P57 and SFRP1 in the aged SVZ, we performed immunofluorescence staining on post-mortem human brain tissue (Supplementary Data 5). In the aged SVZ around 25% of SFRP1^+^ cells in the SVZ expressed SOX2 (Fig. 4c, d). SFRP1 expression is not limited to progenitors, as it is also highly expressed in ependymal cells, cortical neurons (Supplementary Fig. 6a) and OLIG2^+^ cells in the SVZ (Supplementary Fig. 6b, c). While SFRP1 is expressed in the nucleus of progenitors (Fig. 4c) and ependymal cells, it is also expressed in the cytoplasm of neurons (Supplementary Fig. 6a).

SFRP1 inhibits the Wnt pathway by binding to Wnt ligands and by directly binding to β-catenin in the nucleus^42^. To determine whether SFRP1 expression correlated with a quiescent state, we assessed the expression of P57 in SFRP1^+^ cells in the SVZ only. Our results showed that around 78% of the SFRP1^+^ cells in the SVZ expressed P57 (Fig. 4e, f). The majority of P57^+^ cells were positive for SFRP1 (77.79 ± 14.89). This suggests that in the adult SVZ, SFRP1 is mostly expressed by quiescent/primed-quiescent stem cells. We confirmed that SFRP1 is also expressed by post-mitotic progenitors of the fetal human brain at nine gestational weeks (Fig. 4g). Immunofluorescence staining of SFRP1 expression in the SVZ from aged, mid-aged, and fetal post-mortem brain shows an increase in the number of SFRP1^+^ cells from mid-aged (mean age of 61 years) to aged (mean age of 91 years) (Fig. 4h) and from GW9 to GW16-17 (Fig. 4i). We also confirmed in our bulk RNAseq dataset^31^ that *SFRP1* has the highest expression from the SFRP family members, in both CD271^+^ cells and SVZ homogenate isolated from post-mortem brain tissue from healthy donors (Supplementary Fig. 5b).

### SFRP1 inhibition promotes proliferation in iPSC-derived NSC

**Fig. 5:**
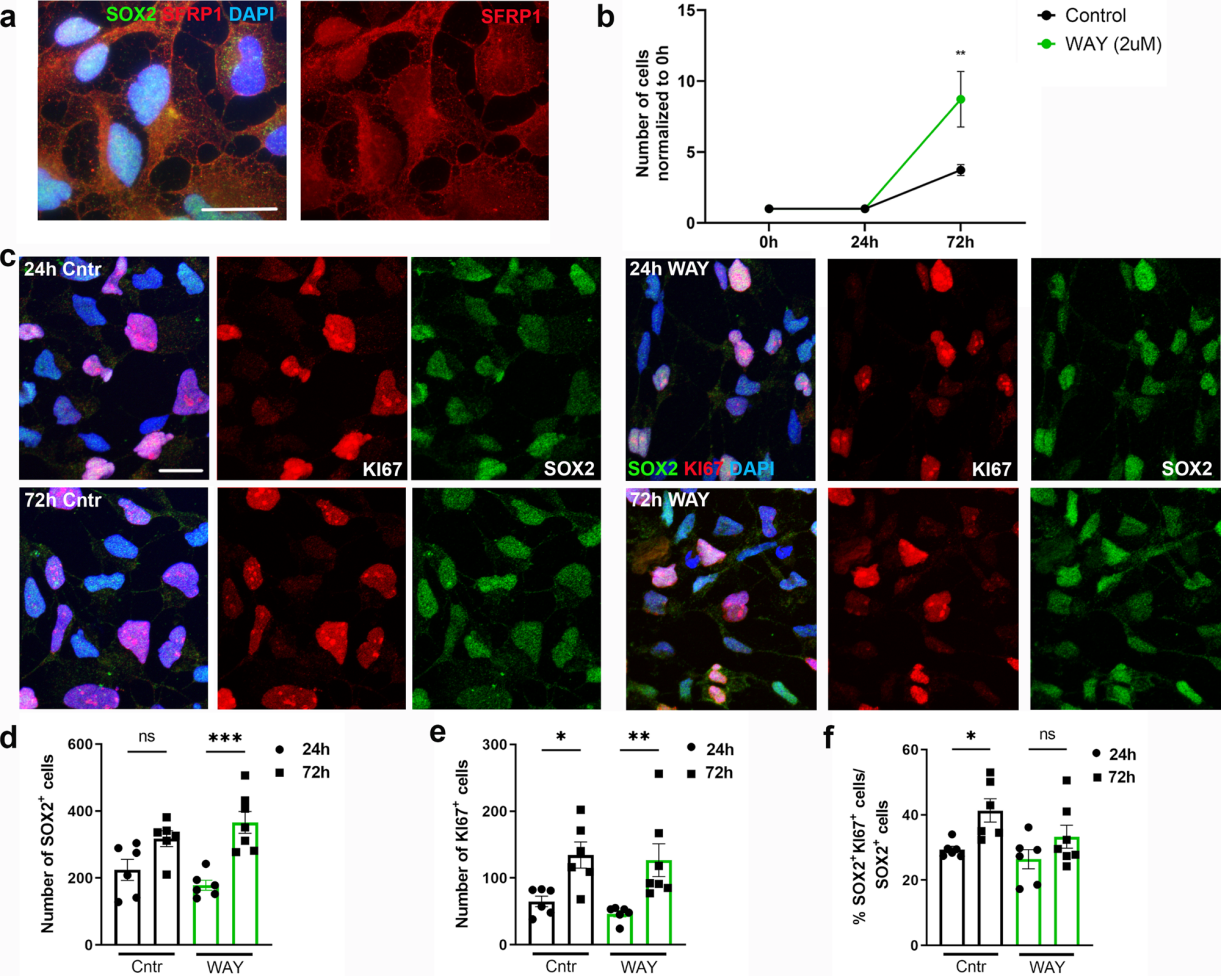
Stimulation of proliferation in iPSC-derived NSCs by inhibition of SFRP1. **a** Representative image of SFRP1^+^ iPSC-derived NSCs showing both nuclear and cytoplasmic labeling. **b** Quantification of the number of cells in control and stimulated condition over time (*n* = 3 biological replicates), **P = 0.0029, two-way ANOVA with Sidák multiple comparisons test. **c** Representative images of SOX2^+^KI67^+^ iPSC-derived NSCs. **d**–**f** Quantification of the number of SOX2^+^ cells (*n* = 6 images per condition), ns = 0.0529, ***P = 0.0001 (**d**), KI67^+^, *P = 0.0218, **P = 0.0061 (**e**) and, percentage of proliferating SOX2^+^ cells, *P = 0.0358, ns = 0.3093 (**f**). Data presented as mean ± SEM. One-way ANOVA with Sidák multiple comparisons test. ns not significant, Cntr control, h hour. Scale bar = 20 µm. Source data are provided as a Source Data file.

A previous study showed that proliferation and differentiation increases during early corticogenesis in Sfrp1^−/−^ mouse embryos^40^. Therefore, we assessed the effect of inhibiting SFRP1 function on proliferation of human NSCs by using a human iPSC-derived neural stem cell line to model human NSCs in vitro. This was done with the small molecule WAY-316606, which is known to sequester SFRP1 in vitro. This molecule prevents SFRP1 from binding to Wnt ligands, thereby stimulating the Wnt pathway^43^. We first confirmed the expression of SFRP1 protein in human iPSC-derived NSCs (Fig. 5a). Most cells expressed SFRP1 protein in the cytoplasm and nucleus, while in some cells cytoplasmic expression was absent. Sequestration of SFRP1, stimulated proliferation of iPSC-derived NSCs 72 h after stimulation in vitro (Fig. 5b–e). This effect is dosage-dependent (Supplementary Figure 7). Stimulation with WAY increased the number of cells by two fold (Fig. 5b). While we observed an increase in SOX2^+^ cells, the percentage of KI67^+^ iPSC-derived NSCs did not increase when compared to control condition (Fig. 5d–f). Our data therefore, suggest that this increase in cell number is mediated by a shortening of the cell cycle rather than an increase in cell activation. This can be explained by the fact that iPSC-derived NSCs do not exit the cell cycle, and instead remain actively cycling. To confirm that the observed effect is mediated by increased activity of the canonical Wnt pathway we performed a Topflash luciferase reporter assay on HEK293 cells. Our results confirmed that the small molecule WAY-316606 activates the canonical WNT pathway through inhibition of SFRP1 (Supplementary Fig. 8). WAY-316606 acts specifically on SFRP1 and does not activate the Wnt pathway when in presence of SFRP5, an SFRP isoform that promotes NSC quiescence in the mouse SVZ^7^.

### SFRP1 is expressed in the postnatal mouse brain

**Fig. 6:**
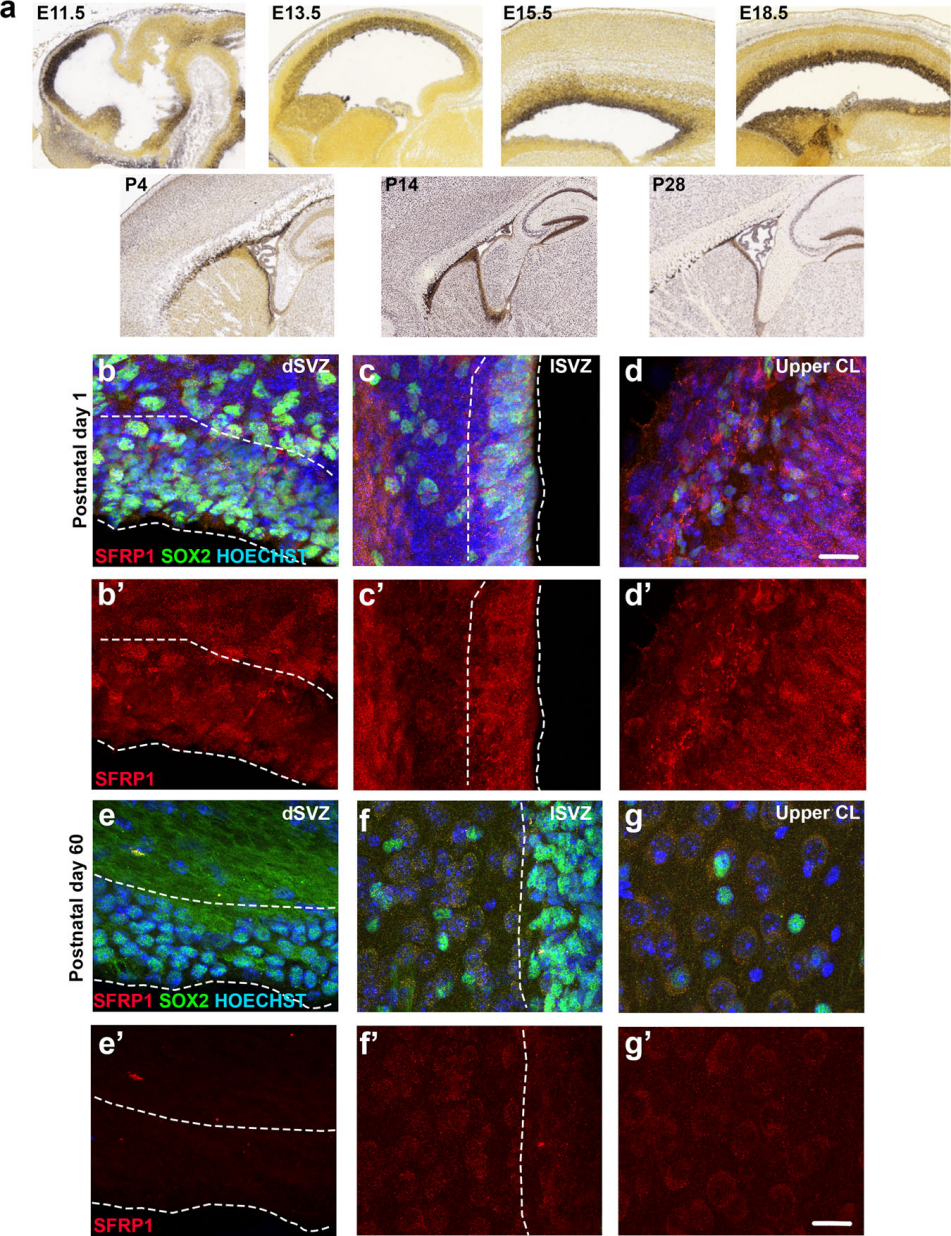
SFRP1 is expressed in the neonatal and adult mouse brain. **a** In-situ hybridization images from the Allen Brain Atlas showing SFRP1 expression in the mouse brain at different developmental stages. **b**–**d** Representative images of SFRP1 expression in the mouse brain at postnatal day 1 (*n* = 3 mice). Examples shown are from the dorsal SVZ (**b**–**b′**), lateral SVZ (**c**–**c′**), and cortical layers 1–2 (**d**–**d′**). **e**–**g** Representative images of SFRP1 expression in a 60 days old mouse brain (*n* = 4 mice; 3 females and 1 male). Examples show dorsal SVZ (**e**–**e′**), lateral SVZ (**f**–**f′**) and upper cortical layer (**g**–**g′**). Hoechst was used as a nuclear counterstaining. E embryonic day, *P* postnatal day, dSVZ dorsal SVZ, lSVZ lateral SVZ, CL cortical layer. Scale bar = 20 µm.

To determine whether SFRP1 inhibition also stimulates proliferation of progenitors in vivo, we first assessed the expression pattern of SFRP1 over time in the embryonic and postnatal mouse brain. In situ hybridization (ISH) data from Allen Brain Atlas showed a gradual increase in *SFRP1* expression from E11.5 to E18.5 (Fig. 6a). During the embryonic period, *SFRP1* is mainly expressed in the germinal regions. Following birth, *SFRP1* expression decreases in the SVZ, while increasing in regions outside the SVZ. We confirmed the ISH data by performing immunofluorescence staining for SFRP1 on P1 and P67 mouse brains. This showed expression of SFRP1 in the SVZ, striatum, and cortex in P1 mouse brain (Fig. 6b–d), and a strong decrease in SFRP1 expression in the SVZ in P67 mouse brains (Fig. 6e–g). Kalamakis et al.^7^ showed that from all members of the SFRP family, only *SFRP5* expression increased with time in the mouse SVZ, while *SFRP1* expression decreased (Supplementary Fig. 5c). Thus, in contrast to the expression pattern of SFRP1 in the human SVZ, its expression is highest in the early postnatal mouse SVZ.

### Inhibition of SFRP1 stimulates the Wnt and Notch pathways

**Fig. 7:**
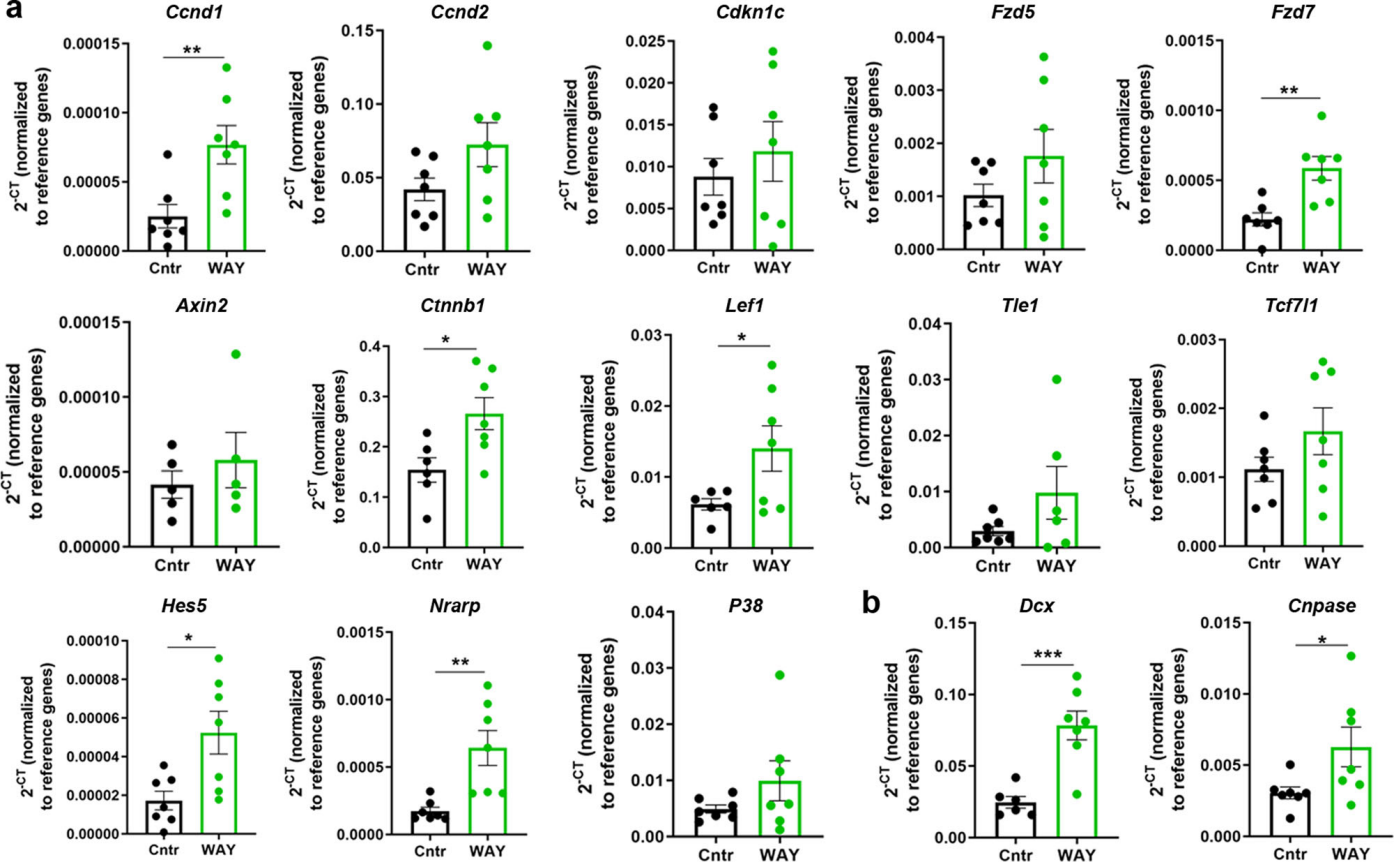
Wnt and Notch pathways are activated after SFRP1 inhibition. **a** RT-PCR analyses of members of the Wnt and Notch signaling pathway 72 h after control or WAY treatment. *Ccnd1*, *P* = 0.0079; *Ccnd2*, *P* = 0.0958; *Cdkn1c*, *P* = 0.4812; *Fzd5*, *P* = 0.2015; *Fzd7*, *P* = 0.0025; *Axin2*, *P* = 0.4514; *Ctnnb1* *P* = 0.0203; *Lef1* *P* = 0.0497; *Tle1*, *P* = 0.1491; *Tcf7l1*, *P* = 0.1737; *Hes5* *P* = 0.0131; *Nrarp* *P* = 0.0041; *P38*, *P* = 0.1938. **b** RT-PCR analyses of *Dcx* and *CNPase* 72 h after control or WAY treatment. *Dcx*, *P* = 0.0007; *CNPase*, *P* = 0.0466. *n* = 7 mice per group, P6; *Ctnnb1, Lef1, Axin2*, and *Dcx* had one outlier removed from control group, Grubbs test with *ɑ* = 0.05. Normalized against the levels of *β-actin* and *Gapdh* reference genes. Data presented as mean ± SEM. Two-tailed Unpaired Student’s *t* test. *P < 0.05; **P < 0.01; ***P < 0.001. Source data are provided as a Source Data file.

Previous studies showed that SFRPs are multifunctional proteins that regulate both Wnt and Notch signaling^40,42^, through which they regulate dopamine neuron development^44^ and cortical expansion^40^. We first assessed whether inhibiting SFRP1 function increased activation of the Wnt and Notch pathways in vivo. SFRP1 was prevented from binding to Wnt ligands by the administration of the small molecule WAY-316606 to two days old mouse pups. We assessed this in the early postnatal mouse brain, as SFRP1 levels are highest in the SVZ at this age (Fig. 6 and Supplementary Fig. 5c). The entire SVZ was dissected 72 h after treatment with WAY-316606 for RT-PCR analysis, focusing on Wnt and Notch pathway-related genes. Our results show a 3.5-fold increase in *Cyclin d1* (*Ccnd1*) (*P* = 0.0079), which promotes cell proliferation^45^ (Fig. 7a). *p57* (*Cdkn1c*) expression did not change (*P* = 0.4812). Moreover, some key genes of the Wnt signaling (*Fzd7* *P* = 0.0025, *Ctnnb1* *P* = 0.0203, and *Lef1* *P* = 0.0497) and the Notch signaling (*Hes5* *P* = 0.0131, and *Nrarp* *P* = 0.0041) were also increased following administration of the small molecule WAY-316606 (Fig. 7a). Administration of WAY also enhanced the expression of *Dcx* (*P* = 0.0007) and *CNPase* (*P* = 0.0466) genes, suggesting increased specification towards neuronal and OPC lineages (Fig. 7b).

### SFRP1 inhibition increases activation of SVZ progenitors

**Fig. 8:**
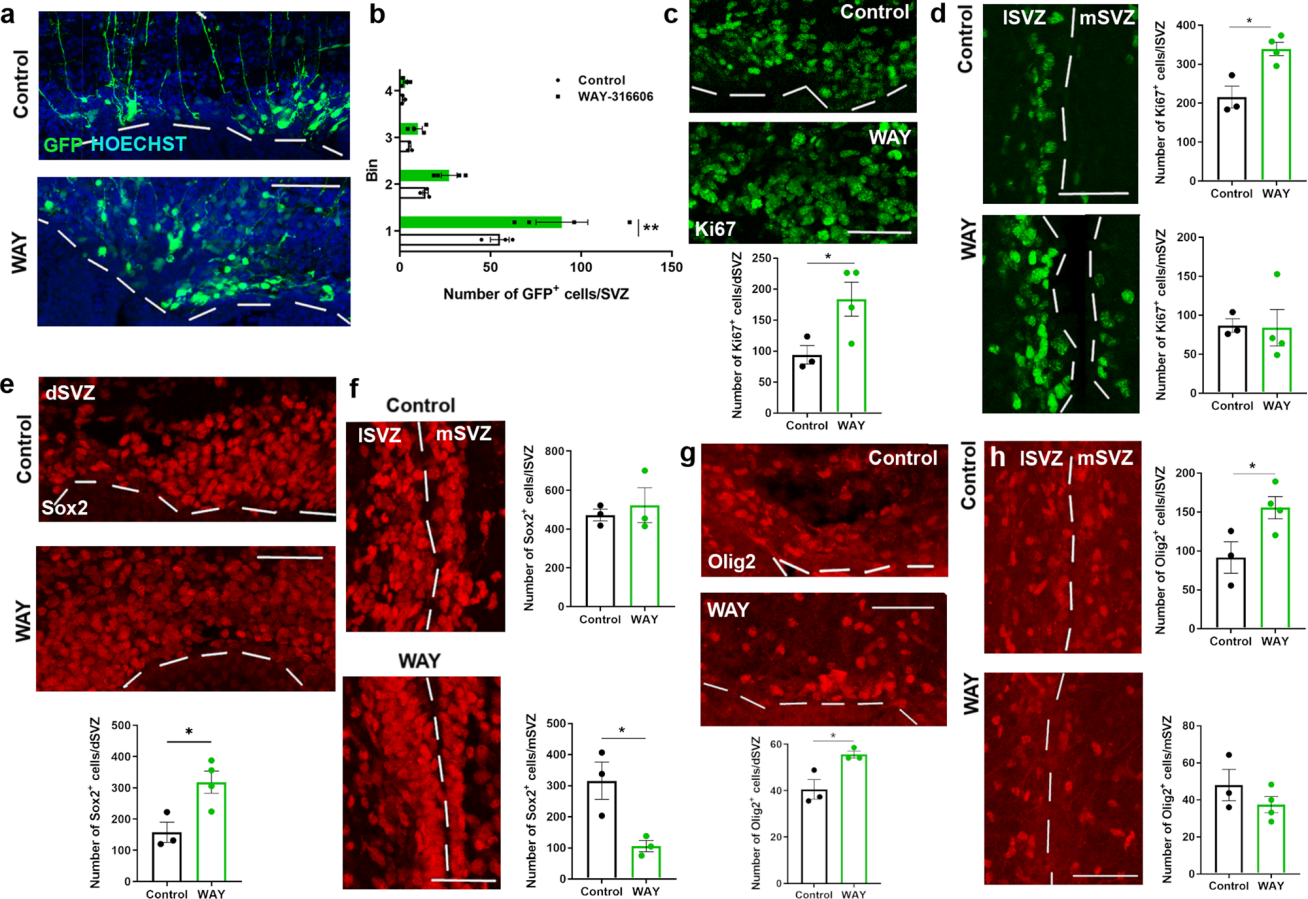
Sequestration of SFRP1 increases activation of NSCs in the early postnatal mouse SVZ. **a**, **b** Representative images of GFP labeled NSCs in the dorsal SVZ (**a**). Increase in GFP^+^ cells in the dorsal SVZ following WAY administration. The region analyzed was divided into four equidistant bins, with bin one corresponding to the SVZ and bin four the area below cortical layer 6. **P = 0.0056, two-way ANOVA with Sidáks multiple comparisons test. *n* = 3 mice (Control) and *n* = 4 mice (WAY), P6. **b**. **c**, **d** Representative images and quantification of the number of Ki67^+^ cells in the dorsal SVZ, *P = 0.0490, *n* = 3 mice (Control) and *n* = 4 mice (WAY), P6. (**c**), lateral, *P = 0.0106, and medial SVZ, *P* = 0.9260, *n* = 3 mice (Control) and *n* = 4 mice (WAY), two-tailed unpaired Student’s *t* test (**d**) after control or WAY administration. **e**, **f** Representative images and quantification of the number of Sox2^+^ cells in the dorsal SVZ, *P = 0.0241 *n* = 3 mice (Control) and *n* = 4 mice (WAY) (e), lateral, *P* = 0.6187, and medial SVZ, *P = 0.0280, *n* = 3 mice (Control) and *n* = 3 mice (WAY), P6, two-tailed unpaired Student’s *t* test (**f**) after control or WAY administration. **g**, **h** Representative images and quantification of the number of Olig2^+^ cells in the dorsal SVZ, *P = 0.0280, *n* = 3 mice (Control) and *n* = 4 mice (WAY) one outlier removed with Grubbs test with alpha = 0.05 (**g**), lateral, P* = 0.0439 and medial SVZ, *P* = 0.2813, *n* = 3 mice (Control) and *n* = 4 mice (WAY), P6, two-tailed unpaired Student’s *t* test (**h**) after control or WAY administration. dSVZ dorsal SVZ, lSVZ lateral SVZ, mSVZ medial SVZ. Data presented as mean ± SEM. Scale bar = 50 µm. Source data are provided as a Source Data file.

We next determined whether WAY-316606 administration would stimulate progenitor proliferation also in vivo. To determine if inhibiting the function of SFRP1 increases the number of GFP^+^ cells and their migration away from the SVZ, we specifically labeled progenitors from the dSVZ by dorsal electroporation of a GFP plasmid at P2 and terminated the pups 72 h after administration of WAY-316606. Our results show a 1.6-fold increase in the number of GFP^+^ cells in the dSVZ (Fig. 8a, b). We did not see a significant increase in migration towards the cortex. The increase in the number of GFP^+^ cells in the dSVZ correlated with a 2-fold increase in proliferating cells in the dSVZ (Fig. 8c). There was also a 1.6-fold increase in Ki67^+^ cells in the lSVZ (Fig. 8d). Our results show that while the number of Sox2^+^ progenitors remains constant in the lSVZ, and increases with 2-fold in the dSVZ, there was a 3-fold decrease in the mSVZ after administration of WAY-316606 (Fig. 8e, f). There was a significant increase in the number of Olig2^+^ cells in both the dorsal and lateral SVZ (Fig. 8g, h).

## Discussion

Although NSCs are present in the adult human SVZ, few neurons are generated after birth^2,4^. NSCs of the rodent SVZ become increasingly quiescent during aging. Studies in rodents suggest that NSC quiescence is regulated by both intrinsic and extrinsic factors (e.g. inflammatory signaling in the SVZ)^7,8,14–23^. The molecular mechanisms that maintain progenitors of the adult human brain quiescent are still unclear. Here, we identify SFRP1, an inhibitor of the canonical Wnt pathway, as a potential target to stimulate progenitor proliferation and differentiation in the adult human SVZ. We show both in vitro, in a human iPSC-derived NSC line, and in vivo, in mice, that inhibiting SFRP1 function with the administration of WAY-316606 increases activation of progenitors, likely by stimulating the activity of both Wnt and Notch pathways. Our work identifies the Wnt antagonist SFRP1 as a potential signal that maintains quiescence of progenitors of the aged human SVZ.

To characterize progenitors from the aging human SVZ, we obtained tissue from donors without neurological or psychiatric disease, which was confirmed by a thorough pathological assessment. This is important to avoid confounding factors due to changes in the neurogenic niche environment because of disease-related mechanisms. As expected, the assessment also showed amyloid β deposition and neurofibrillary degeneration to varying degrees in the donors. Although we show that there is no segregation of cells by donor, the differences in degree of pathology and in age, should be kept in mind and might have influenced the number of progenitors that could be isolated, as well as their gene signature.

Interestingly, we show that progenitors from the adult human SVZ are primed towards the oligodendroglial lineage, as genes from this lineage are highly expressed, while canonical markers of the neuronal lineage are practically absent. Integration of our data with published datasets from the fetal forebrain^38^ and adult white matter^39^, revealed the CD271^+^ cells to be late OPCs. We cannot exclude that CD271 may label a subpopulation of progenitors in the human SVZ. It is likely that human progenitors of the SVZ are heterogeneous as in the rodent SVZ, where progenitors differ in their lineage specificity depending on their location within the SVZ^46,47^. A previous study suggested that CD271 is expressed specifically in OPCs following demyelinating brain injuries in both humans and rodents^48^. Therefore, we cannot conclude, based on our current results, that either early OPCs or NSCs are absent from the aged human SVZ.

The turnover rate of oligodendrocytes stabilizes around five years of age and remains low throughout the human lifespan^49^. Our results indicate that this low turnover rate is not caused by the absence of OPCs in the SVZ, but rather due to increased quiescence. Here, we identified SFRP1, a Wnt pathway antagonist, as a possible signal that maintains late OPCs in a quiescent state in the human SVZ. SFRP expression is not restricted to NSCs, but is also expressed by astrocytes^50^ and microglia^51^. Two other members of the SFRP family, SFRP3 and SFRP5, were shown to regulate NSC quiescence in the mouse brain. SFRP3 maintains NSCs in a quiescent state in the dentate gyrus^52^ of adult mice and its deletion increases NSC activation and maturation. SFRP5 was shown to maintain NSCs of the aged mouse SVZ quiescent and when blocked by the administration of antibodies, activation of aged NSCs was increased^7^. Neither SFRP3 nor SFRP5 are expressed in our datasets (Supplementary Figure 5)^31^, suggesting species-specific differences in expression profile. Indeed, our data show that while in humans, SFRP1 expression in the SVZ increases with age, its expression decreases in young adult mice. Hence, SFRP1 could be the human homolog of SFRP5 in regulating NSC quiescence in the aged human SVZ.

The mechanisms through which SFRPs modulate the canonical Wnt pathway to maintain cells in a quiescent state are still unclear. Growing evidence suggests that members of the SFRP family function as tumor suppressor genes, as they are lowly expressed in different types of tumors, including meduloblastoma^53–55^. Methylation of the promoter region of *SFRP*, results in its decreased expression, which correlates to increased malignancy^54^. Indeed, low levels of SFRP1 have been shown to increase proliferation in different tumor cell lines^53^. A recent study proposed a Wnt-independent mechanism in which nuclear SFRP1, 2, and 5 directly bind to β-catenin, thus inhibiting its transcriptional activity and the expression of cancer stem cell related genes^42^. We show that inhibition of SFRP1, by the administration of WAY-316606, stimulates proliferation and differentiation of NSCs both in vitro and in vivo by activation of the canonical Wnt pathway. The luciferase Topflash reporter assay also shows that WAY-316606 acts specifically on SFRP1 and does not activate the canonical Wnt pathway when in the presence of SFRP5. These results are also supported by a study where bone formation was stimulated through sequestration of SFRP1 with WAY-316606^56^ showing that WAY-316606 inhibits SFRP1 activity with 40%, while SFRP2 and SFRP5 activities were only decreased by 2–5%. All together, these data suggest that the small molecule WAY-316606 promotes the activity of the canonical Wnt pathway through inhibition of the Wnt antagonist SFRP1.

In conclusion, our work identifies SFRP1 as a potential signal that maintains progenitors of the aged human SVZ in a quiescent state, supporting the possibility to re-activate progenitors of the aged human brain to regenerate the brain following injury or neurodegenerative diseases.

## Methods

### Animals

All animal experiments were performed in accordance to the international guidelines from the EU directive 2012/63/EU and approved by the Experimental Animal Committee Utrecht (University Utrecht, Utrecht, Netherlands) (CCD number: AVD1150020184944). Animal experiments were carried out on wild-type C57BL/6j mice (Charles River, The Netherlands) aged 1 (P1), 2 (P2), or 60 (P60) days. Both males and females were used. The morning when a plug was observed is considered as E0.5 and the day of birth is defined as P0. Mice were housed in groups, with access to food and water ad libitum on a 12 h light/dark cycle, humidity of 45–65% and a temperature between 20 and 24 °C. Handling of pups was kept to a minimum to reduce stress to both mom and pups.

### Human post-mortem brain tissue for single-cell RNAseq

Fresh human post-mortem dorsal SVZ including adjoining white matter tissue (*n* = 3 donors) (Supplementary Fig. 1a) was obtained from donors without known neurological or psychiatric disease from the Netherlands Brain Bank (NBB; https://www.brainbank.nl), Netherlands Institute for Neuroscience, Amsterdam. The NBB performs quick brain autopsies to ensure high tissue quality. All donors have given informed consent to the NBB to perform autopsies for tissue isolation, access to medical records for research purposes and consent to publish clinical information potentially identifying individuals. The independent Medical Ethics Committee of the VU University Medical Center has reviewed and agreed to the procedures of the NBB concerning donation of brain material for scientific research (2009/148) (https://www.brainbank.nl/about-us/the-nbb/). Autopsies are performed by the NBB at the designated premises of the VU Medical Center in Amsterdam, the Netherlands. The NBB adheres to the standards for quality, safety and ethics for obtaining and handling of human tissue, as described in BrainNet Europe’s Code of Conduct for brain banking. This study was reviewed and authorized by NBB’s scientific committee. The study design and conduct complied with all relevant regulations regarding the use of human study participants and was conducted in accordance with the criteria set by the Declaration of Helsinki. This study was performed according to the Dutch and European legal and ethical regulations. To ensure donor anonymity only an autopsy serial number, which is given by the NBB, is disclosed. This number contains the year that the autopsy was performed and the number of the autopsy. Directly after autopsy, samples are placed in Hibernate-A medium (ThermoFisher Scientific, Landsmeer, The Netherlands, A1247501) and were kept cold until isolation. Samples had a mean post-mortem delay of 6.35 h (Supplementary Data 1).

### Human post-mortem brain tissue for immunofluorescence

Adult post-mortem dorsal SVZ tissue from donors without known neurological disease was obtained from the NBB (Supplementary Data 5) (*n* = 5 donors). All donors have given informed consent to the NBB to perform autopsies for tissue isolation, access to medical records for research purposes and consent to publish clinical information potentially identifying individuals. The independent Medical Ethics Committee of the VU University Medical Center has reviewed and agreed to the procedures of the NBB concerning donation of brain material for scientific research (2009/148). The NBB adheres to the standards for quality, safety, and ethics for obtaining and handling of human tissue, as described in BrainNet Europe’s Code of Conduct for brain banking. This study was reviewed and authorized by NBB’s scientific committee. The study design and conduct complied with all relevant regulations regarding the use of human study participants and was conducted in accordance with the criteria set by the Declaration of Helsinki. This study was performed according to the Dutch and European legal and ethical regulations. To ensure donor anonymity only an autopsy serial number, which is given by the NBB, is disclosed. Material was fixed in formalin and embedded in paraffin. Fetal human brain tissue was obtained from abortion material without developmental structural chromosomal abnormalities (Supplementary Data 6) from the Chinese University of Hong Kong. Forebrain tissue was obtained from gestational week (GW) 9 (*n* = 3 donors), GW 16 (*n* = 2 donors), and GW 17 (*n* = 2 donors), fixed in 4%-paraformaldehyde (PFA) and embedded in paraffin. Donors of fetal tissue have given informed consent to the use of the tissue for research. Women being admitted for pregnancy termination due to different clinical indications were invited to donate their conception material, including placenta, abortus and fetal blood for study. The participants understood that the fetal tissue would normally be discarded as medical waste. Participation was entirely voluntary, without compensation, and the decision of the donor did not interfere with the clinical care. Fetal tissue samples without structural and chromosomal abnormalities, were collected after the completion of the termination of the pregnancy. Involvement in the study did not confer any additional risk over the routine clinical treatment. The participants had the right to withdraw from the study and request for the collected samples to be destroyed at any time. All clinical information will remain confidential. Identity of the donor is kept anonymous by the use of a serial number. This study was performed according to the Dutch, European, and Hong Kong institutional ethical regulations for the use of abortion material. This study was approved by the Chinese University of Hong Kong – New Territories East Cluster Clinical Research Ethics Committee (CREC), Faculty of Medicine, The Chinese University of Hong Kong (under the human ethics approval reference number CREC-2004.330). The study design and conduct complied with all relevant regulations regarding the use of human study participants and was conducted in accordance with the criteria set by the Declaration of Helsinki.

### Single-cell RNA sequencing

Following single-cell sorting the plate was centrifuged at 300×*g* for 1 min and kept at −20 °C until further processing. Sort-seq was run on single-cells as described in Muraro et al., 2016^57^, which is based on single-cell RNA sequencing by multiplexed linear amplification (Cel-Seq2 protocol from Hashimshony et al.^32^). Cells were lysed for 5 min at 65 ^o^C, followed by dispersion of RT and second strand mixes with the Nanodrop II liquid handling platform (GC Biotech, Waddinxveen, NL). After in vitro transcription, the cDNA library was prepared according to the Cell-Seq2 protocol. The primers used consisted of 24 bp polyT stretch, a 6 bp random molecular barcode (UMI), a cell-specific 8 bp barcode, the 5′ Illumina TruSeq small RNA kit adaptor (all from Illumina, San Diego, CA, USA) and a T7 promoter (ThermoFisher Scientific, Ambion AMB13345). TruSeq small RNA primers (Illumina) were used for making the Illumina sequencing libraries. Sequencing was done on the Illumina NextSeq 500 platform by sequencing paired-end at 75 bp read length (25 bp from R1 and 50 bp from R2) with a sequencing depth of 15M reads per 384-well plate.

### Luciferase reporter (Topflash) assay

HEK293T cells (LGC Standards, Middlesex, UK, ATCC-CRL-11268) were grown in a 96 well plate and transfected 8 h after plating with 33 ng Topflash (Addgene TA viral promotor, Watertown, MA, USA, 12456) and 3,3 ng Renilla (pRL-TK Renilla, Promega, Leiden, The Netherlands) using PEI transfection reagent (polyethylenimine, linear, 25000mw, Polysciences 23966-2, Bergstrasse, Germany). 24 h after transfection, HEK293T cells were incubated with: (i) 1% CHAPS control; (ii) recombinant human Wnt3a (Wnt3a: 180 ng/mL, R&Dsystems 5036-WN/CF); recombinant human Wnt3a with recombinant human SFRP5 protein (5 µg/mL, Thermo Fisher Scientific 15953709); (iii) recombinant Wnt3a with recombinant human SFRP1 protein (5 µg/mL, Peprotech 120-29, London, UK); (iv) recombinant Wnt3a with recombinant SFRP1 protein and small molecule (2 µM WAY-316606); and (v) recombinant SFRP5, recombinant Wnt3a and WAY small molecule. After 24 h, cells were harvested and Luciferase activity was determined according to the manufacturer’s manual (Dual-Luciferase Reporter Assay System, Promega, Madison, WI, USA, E1910). Values are presented as Firefly activity normalized to Renilla values and corrected for the 1% CHAPS control.

### Immunofluorescence staining

Immunofluorescence was performed on human post-mortem fetal and adult SVZ tissue (Supplementary Data 5 and Supplementary Data 6). 7 µm thin paraffin sections were deparaffinized, washed in PBS and washed in 3% hydrogen peroxide solution for 10 min to block endogenous peroxidase. Antigen retrieval was performed in 10 mM citrate buffer pH 6.0 at 80 °C for 20 min. After cooling down sections were thoroughly washed in 0.25% Triton-X100 in PBS, and incubated with blocking solution 2% BSA with 0.4% Triton-X100 in PBS at room temperature for 1 h. Sections were incubated with primary antibodies at 4 °C overnight. The following primary antibodies were used: rabbit anti-SOX2 (1:100; EMDMillipore, Burlington, MA, USA, NBP1-20136, lot Q2922255), goat anti-SOX10 (1:200; R&Dsystems, Minneapollis, MN, USA, AF2864), goat anti-SFRP1 (1:100; R&Dsystems, AF1384, lot IRQ061610A), mouse anti-PCNA (1:100; Santa Cruz, Dallas, TX, USA, sc-56), rabbit anti-P57 (1:100; Abcam, Burlingame, CA, USA, ab75974) and rabbit anti-OLIG2 (1:100; IBL, Minneapolis, MN, USA, 18-953, lot 1B-327). After washing, sections were incubated with the corresponding horseradish peroxidase antibody (1:500; Jackson ImmunoResearch, UK) for 1 h at room temperature, washed, followed by incubation with Tyramide Signal Amplification (Perkin Elmer, Waltham, USA, NEL704A001 and NEL701A001) Cy3 (1:50) for 10 min, or with Fluorescein (1:300) for 5 min. Sections were washed and incubated with TrueBlack for 30 sec (Biotium, CA, USA, 23007) to quench autofluorescence and immediately washed in PBS. Nuclear counterstaining was done with Hoechst 33258 (1:5000; Invitrogen, 94403) and sections were mounted with FluorSave (EMD Millipore, 345789).

Mice were sacrificed with an overdose of pentobarbital and fixed by transcardial perfusion with PBS followed by 4% PFA (wt/vol). Brains were dissected and post-fixed in 4% PFA at 4 °C. Free floating vibratome serial sections were cut at a thickness of 40 µm. Sections were blocked in 2% BSA with 0.4% Triton-X100 in PBS at room temperature for two hours and incubated with primary antibodies diluted in 2% BSA with 0.25% Triton-X100 in PBS at 4 °C overnight. The following primary antibodies were used: rabbit anti-Sox2 (1:500; EMDMillipore), rabbit anti-Sfrp1 (1:500; Abcam, ab4193), mouse anti-Ki-67 (1:1000; Abcam, ab15580), and rabbit anti-Olig2 (1:200; IBL). After thorough washing, sections were incubated with the corresponding secondary antibodies conjugated to Alexa-555 or Alexa-647 (1:1000; Invitrogen) at room temperature for two hours. Nuclear counterstaining was done with Hoechst 33258 (1:5000; Invitrogen) and sections were mounted with FluorSave (EMD Millipore).

### qRT-PCR

Following RNA extraction with Trizol and chloroform, RNA was precipitated in isopropanol. Reverse transcription of mRNA was done using the Quantitect Reverse Transcription kit (Qiagen GmbH, Hilden, Germany, 205311) according to the manufacturers’ protocol. Primers were designed using the Primer-BLAST designing tool from the NCBI website (https://www.ncbi.nlm.nih.gov/tools/primer-blast/; listed in Supplementary Data 7) and from Furutachi et al.^9^, Kase et al.^58^, Esteve et al.^40^, and Bonnefont et al.^59^. qRT-PCR was performed on the Quantstudio 6 Flex (Applied Biosystems, Life Technologies) using FastStart Universal SYBR green master (Rox) reagent (Roche Diagnostics GmbH, 04913914001) and analyzed using the Quantstudio Realtime PCR software (version v1.1; Applied Biosystems). Results are shown as 2^-Ct^ values normalized to the reference genes *Gapdh* and *β-actin*.

### Statistical analysis

Data shown in Figs. 4, 5, 7, 8 and Supplementary figs. 6–8 are expressed as mean ± SEM. Measurements were taken from distinct samples. The number of samples analyzed is stated in the figure legend. Significance was tested on GraphPad Prism 7 (La Jolla, Ca, USA) with two-tailed unpaired t-test, one-way ANOVA with Sidák multiple comparisons test or a two-way ANOVA with Sidák multiple comparisons test. A *P*-value of <0.05 was considered statistically significant. Outliers were detected using the Grubbs test with *α* = 0.05.

### Reporting summary

Further information on research design is available in the Nature Research Reporting Summary linked to this article.

## Supplementary information

Supplementary Information

Description of Additional Supplementary Files

Supplementary Data 1

Supplementary Data 2

Supplementary Data 3

Supplementary Data 4

Supplementary Data 5

Supplementary Data 6

Supplementary Data 7

Reporting Summary

## Data Availability

The single-cell RNA sequencing dataset generated in this study has been deposited in NCBI’s Gene Expression Omnibus database under the accession number: GSE164986. The source data generated in this study are provided in the Source Data file. The Zhong et al.^38^ and Jäkel et al.^39^ data used in this study are available in the NCBI’s Gene Expression Omnibus database under accession codes GSE104276 and GSE118257, respectively. Source data are provided with this paper.

## Source data

Source Data

## Materials availability

This study did not generate new unique reagents.

## Code availability

No new code was generated.
