# Supplementary material for: Single-cell profiling of human subventricular zone progenitors identifies SFRP1 as a target to re-activate progenitors
Source: Nat Commun. 2022 Feb 24;13:1036. doi: 10.1038/s41467-022-28626-9 (PMC8873234; doi:10.1038/s41467-022-28626-9)
Supplement: Supplementary file 1 — Supplementary Information [file 41467_2022_28626_MOESM1_ESM.pdf]

Single-cell profiling of human subventricular zone progenitors identifies SFRP1 as a target to re-activate progenitors

Supplementary methods

Tissue dissociation and single-cell isolation

Progenitors, astrocytes, and microglia were isolated as described in Donega et al.[1]. Fresh dorsal SVZ tissue was dissociated mechanically followed by enzymatic dissociation with 0.3% trypsin (Gibco, Life Technologies, Paisley, UK, 11538876) and 20 U/ml DNaseI (Roche Diagnostics GmbH, Mannheim, Germany, 4536282001) at continuous shaking (70 rpm on an Incu shaker mini) at 37°C for 30 min. Fetal bovine serum (FBS) (Thermo Fisher Scientific, Hampton, NH, USA, A31605) was added to the tissue homogenate to inhibit the enzymes and the homogenate was centrifuged for 10 min at 650 x g. Following two washes in GKN-BSA buffer (0.22% glucose and 0.33% bovine serum albumin dissolved in PBS). Tissue homogenate was centrifuged at 650 x g for 10 min and filtered through a 100 µM nylon cell strainer (Corning, New York, USA, CLS431751). Next, Percoll (GE Healthcare Bio-sciences AB, Uppsala, Sweden, GE17-0891-01) density gradient centrifugation (30 min at 3200 x g at 4ºC) was used to remove myelin and debris. The turbid phase containing the cellular fraction (second fraction with lowest density) was collected and washed in GKN-BSA buffer. The cellular fraction was then incubated with primary antibodies against rabbit anti-GLT-1 (1:50; R&D systems, NBP1-20136) and Fc-receptor (1:20; Miltenyi, Bergisch Gladbach, Germany, 130-059-901) at 4ºC for 30 min. Following incubation, cell suspension was centrifuged at 300 x g for 10 min and incubated with the following fluorescently conjugated antibodies: donkey anti-rabbit AF488 (1:250; Invitrogen, Carlsbad, CA, USA, R37118), Fc-receptor (1:20; Miltenyi), anti-CD11b-PE (1:80; Invitrogen, 12-0112-82, M1/70 clone) and anti-CD271 APC (1:11; Miltenyi, 130-113-418, clone 5170131291) for 30 min at 4ºC. Cells were passed through a 70 µm filter to remove any clumps or debris before sorting. 7-AAD was used as a marker to exclude dying cells (1:50; BD Pharmingen, AB_2869266). FACS was performed immediately after antibody incubations. See Supplementary figure 1b for FACS schemes.

Postnatal electroporation

pcDNA3-EGFP plasmid (EGFP codon from Clontech, 6085-1) was used at a final concentration of 3 µg/µL. Electroporation of the mouse dorsal SVZ (dSVZ) was performed as described in Boutin et al.[2]. P2 mouse pups (n=7) were anesthetized with a mixture of 5%

isoflurane and oxygen (0.5 mL/min). Pups were fixed on a plate placed in a stereotaxic rig and 2 µL of a mix of the plasmid (final concentration of 3 µg/µL) and Fast Green was injected at the level of the right lateral ventricle at a depth of 1.5 mm from the surface of the skull using a Hamilton syringe with a 34G needle (Nevada, USA). Following injection, mice were immediately subjected to five electrical pulses (85V, 50 ms, separated by 950 ms intervals) using the ECM 830 square wave electroporator and a 7 mm platinum tweezertrode (BTX, Holliston, USA).

Intraperitoneal injection of small molecule

Inhibition of SFRP1 was done by intraperitoneal injections of WAY-316606 (HY-10858 MedChemExpress, NJ, USA), which binds to SFRP1. Intraperitoneal injection was given between P2-P5 for the early postnatal stimulation, twice a day (six injections of 10 µL each), with a final concentration of 0.3 mM (dissolved in sterile PBS with 10% DMSO). Control pups received six injections of PBS with 10% DMSO. An n=3 (control) and n=4 (treated) was used for immunofluorescence analysis. Fourteen pups were terminated (n=7 controls and n=7 treated) and the SVZ was isolated for qRT-PCR analysis. Briefly, the brain was removed and the entire SVZ was dissected and immediately frozen in liquid nitrogen until further use.

Human iPSC-derived NSC culture

Generation and characterization of induced pluripotent stem cells (iPSCs) from donor OH3.1, was described in Harschnitz et al.[3]. iPSCs were cultured at 37 °C with 5% CO2 in feeder free conditions on dishes coated with Geltrex (Thermo Fisher Scientific, A1413202) in StemFlex medium (Thermo Fisher Scientific, A3349401). Passaging was performed using 0.5 mM EDTA (Thermo Fisher Scientific, 15-575-020). Cells were seeded in media supplemented with 5 µM Y27632 rho-kinase inhibitor (ROCKi; Axon, 1661). iPSCs were frequently tested for mycoplasma infection (Lonza, LT07-318).

NSCs were derived from iPSCs as previously described with minor modifications[4,5] based on work by Chambers et al.[6]. iPSCs were seeded at a 3.8×10^4 cells/cm^2 density on Geltrex-coated well plates in StemFlex medium. Induction of the NSC phenotype was started after 24 hours by adding 10 µM of SB-431542 (Axon, 1683) and 2 µM Dorsomorphin (R&D Systems, 3093/10) to knock-out serum replacement (KOSR) medium, consisting of DMEM/F-12 + GlutaMax (Thermo Fisher Scientific, 31331-028) supplemented with 20% KOSR (Thermo Fisher Scientific, 10828028), 1× Non-essential Amino Acids (NEAA; Thermo Fisher Scientific, 11140-076), 1:500 2-mercapthoethanol (Thermo Fisher Scientific, 31350-010), and 1 % pen-strep (ThermoFisher Scientific,15140-122). The medium was

changed on a daily basis. After five days, SB-431542 was removed and the medium was changed with partly KOSR and partly N2/B27 medium, consisting of equal parts DMEM/F-12 + GlutaMax and Neurobasal medium, supplemented with 1:500 2-mercapthoethanol, 1× N2 (Thermo Fisher Scientific, 17504-048), and 1:500 B27 (Thermo Fisher Scientific, 17502-044). This replacement followed set proportions (25%, 50%, 75% and 100%). After 10 days of differentiation, the cells were passaged 1:2 in N2/B27 medium with TrypLe Express (Thermo Fisher Scientific, 12604054) on 0.1 mg/mL PDL (Sigma-Aldrich, P0899) and 1μg/mL laminin (Thermo Fisher Scientific, 23017-015) coated 6-well plates. On day 15, NSC culture condition was initiated. NSC culture medium consisted of DMEM/F-12 + GlutaMax supplemented with 1× N2, 1:1000 B27, 10 ng/mL fibroblast growth factor 2 (FGF2; Thermo Fisher Scientific, AA10-155), 10 ng/mL human epidermal growth factor (EGF; PeproTech, AF100-15), and 1% pen-strep. The medium was changed daily. NSCs were passaged weekly and kept in high-density monolayers (4×10^4 cells/cm^2).

For the proliferation assay, NSCs were seeded at 1.5×10^4 cells/cm^2 in triplicates in a 24-well plate. The cells were treated with 2 μM WAY-316606 (HY-10858)[7] dissolved in DMSO, or DMSO only in the control condition. Cells were stimulated twice: directly after seeding and 48 hours later. The number of cells was determined at 24 hours and 72 hours after stimulation with WAY-316606, i.e. 24 hours and 72 hours after seeding, by live cell counting using Countess II Automated Cell Counter (Thermo Fisher Scientific, A27977) in combination with Trypan blue (Thermo Fisher Scientific, T10282).

Immunocytochemistry

Cells were fixed in 4% formaldehyde (Merck, 47608) for 10 min. After washing multiple times, the coverslips were blocked in PBS containing 0.2% Triton-X100 (Merck, 9036-19-5) and 10% normal donkey serum (NDS; Merck, 566460) at room temperature for 1 hour. Next, coverslips were incubated with primary antibodies (rabbit anti-SOX2, mouse anti-KI67 and, goat anti-SFRP1) diluted in 0.02% Triton-X100 and 1% normal donkey serum at 4°C overnight. After washing several times with PBS, the coverslips were incubated with the appropriate secondary Alexa Fluor 488 or 594 (Invitrogen) antibodies in PBS / 0.02% Tween-20 with DAPI (Sigma, d9542) at room temperature for 1 hour. After washing, the slides were mounted in FluorSave (Merck, 345789).

Images used for analysis were taken with a Zeiss AxioScope A1 epifluorescence microscope. All images were captured with the same exposure time for both conditions. Six images were quantified per condition (n=6) and analyzed in ImageJ v1.53c. An automated

counting method using the Moments threshold for KI67 and SOX2, and the RenyiEntropy threshold for DAPI staining, which is provided by the software, was used to determine the number of cells positive for both markers. High magnification images were taken with a Zeiss AxioScope A1 epifluorescence microscope using 100x/ 1.3NA oil objective, an AxioCam camera (Zeiss), and the software AxioVision v4.8.2.0 or on a Zeiss LSM880 confocal laser microscope using 63x/ 1.4NA oil DIC M27 objectives, an AxioCam MRm camera (Zeiss), and the software Zen black Z.1SP3. Images were taken with a z-step of 1.0 µm and a resolution of 1024x1024.

Quantification and statistical analysis

Alignment and processing

Alignment to the human transcriptome was performed using a custom pipeline (https://github.com/anna-alemany/transcriptomics/tree/master/mapandgo/starmap)[8]. Raw reads were trimmed, aligned to the Ensembl release 75 Homo sapiens genome using RNA STAR version 2.53A (Spliced Transcripts Alignment to a Reference)[9] and demultiplexed using cell specific barcodes. Reads that mapped equally well to multiple locations were discarded. Duplicate reads were removed in the case of identical combinations of library, cellular, and molecular barcodes and were mapped to the same gene. Transcript count was adjusted based on read counts and the presence of one of the 4096 possible UMI's.

Filtering and normalization

All analysis were performed on R-studio version 4.0.2. Quality check and filtering was performed on Seurat v3.2.2[10] using the following parameters: Only genes that were detected in at least two cells were taken for downstream analysis. Cells that had less than 100 genes or more than 3000 genes detected and that had more than 6% of their counts mapped to the mitochondrial genome were removed. This resulted in a Seurat Object of 728 cells and 22682 genes.

Normalization was done using Seurat (NormalizeData function with LogNormalize method) where a generalized linear model for each gene is constructed[11]. The effect of differences in sequencing depth, library preparation, and donor was removed from the normalized expression values with the ScaleData function from Seurat.

Single-cell clustering and visualization

Highly variable genes were identified using Seurat v3.2.2 (FindVariableFeatures function). Clustering was then performed by first reducing dimensionality of the dataset by running a

principle component analysis (PCA). Number of principle components was selected based on heatmap (DimHeatmap function), Jackstraw plot (JackStrawPlot function) and Elbow plot (ElbowPlot function). The top 14 PC's were used to find clusters (FindNeighbors function and FindClusters function with the resolution set to 0.8). For visualization of the clusters, Uniform Manifold Approximation and Projection (UMAP) coordinates were calculated in PCA space using Seurat (RunUMAP function). UMAP plots were then colored by cluster identity, cell origin, or gene expression values (FeaturePlot function). Marker genes were determined using the FindMarkers function. Genes were considered marker genes when it was expressed in more than 25% of the cells with a logFC threshold of at least 0.25 (natural log). The Wilcoxon rank sum test was used to identify marker genes. P-value adjustment was performed using the Bonferroni correction.

Gene ontology enrichment analysis

Gene Ontology (GO) analysis was performed on cluster marker genes with adj P-value < 0.01 (Supplementary Data 2) with the GO biological process using the Enrichr web-based tool[12,13] (http://amp.pharm.mssm.edu/Enrichr/).

Integration of single-cell datasets

Integration of single-cell datasets published in Zhong et al.[14] and Jäkel et al.[15] with our dataset was done using Seurat v3.2.2 as described in Stuart et al.[16]. This pipeline allows the comparison of multiple datasets by identifying in each dataset the 2000 most variable genes using the FindVariableGenes function. Assuming that there are similarities between datasets and that a subset of cells have a shared biological state, a set of molecular features could be identified. These so-called "anchors" were determined with the FindIntegrationAnchors function with dims set on 35. These anchors are then passed to the IntegratedData function, which creates a Seurat object. This object contains an integrated expression matrix of all the cells, which will then cluster by cell type instead of platform technology or species.

Pseudo-time analysis

Genes whose expression changes over time were identified by using Monocle3 v0.2.3.0[17,18]. Every gene was fitted in a linear regression model. Multiple hypothesis testing was corrected using the Benjamini and Hochberg test. Genes that had a time-dependent expression were filtered. Differentially expressed genes were considered significant when q-value < 0.01.

Imaging and quantification

The stained sections were analyzed on a Zeiss LSM880 confocal laser microscope using 40x/ 1.3NA oil DICII objectives (EC PlnN), an AxioCam MRm camera (Zeiss), and the software Zen black Z.1SP3. Images were taken with a z-step of 2.5 µm and a resolution of 1024x1024. For staining of fetal forebrain sections two to four images were quantified. For the quantifications on the human SVZ, around 50% of the progenitors present in one SVZ section was imaged and analyzed. For mouse staining, the entire dorsal, medial, and lateral walls from the SVZ were quantified on at least two brain sections to avoid biases related to regional heterogeneity. The number of GFP positive cells was quantified within the SVZ (bin number 1) and from the border of the SVZ until below cortical layer 6 (bins 2-4). Because both frontal and caudal SVZ sections were quantified, the size of bins 2-4 were determined based on the distance from the SVZ to the subplate layer, which was divided by three to ensure equal bin sizes within frontal and caudal sections. Cells were quantified using the cell counter plugin from Fiji (version 1.52p)[19].

Supplementary Figures

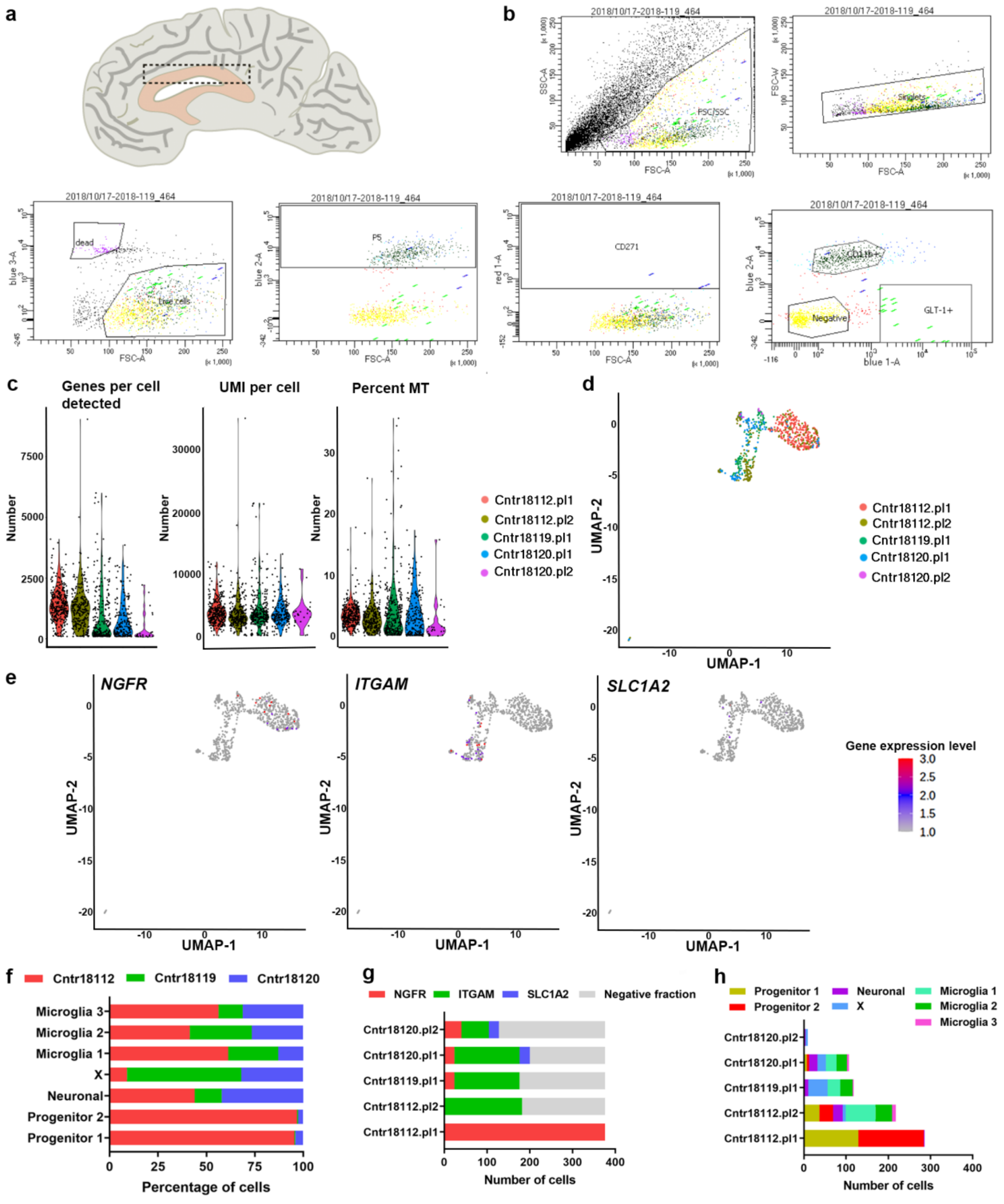

Supplementary Figure 1. **Experimental design and validation of FACS-sorting protocol. a** The SVZ isolated contained the frontal and caudal dorsal SVZ below the corpus callosum. **b** FACS scheme to isolate progenitors, astrocytes, and microglia. **c** Violin plots showing number of genes detected per cell, UMI per cell, percentage MT (mitochondrial) genes per sequenced plate (pl). Cntr18112: 504 cells, mean UMI per cell 3935, mean gene number per cell 1352, Cntr18119: 118 cells, mean UMI per cell 4181, mean gene number per cell 873, Cntr18120: mean UMI per cell 3991, mean gene number

per cell 779. **d** UMAP showing the origin of individual cells by donor number and plate. **e** Feature plots showing the expression of *NGFR* (i.e. CD271), *ITGAM* (i.e. CD11b), and *SLC1A2* (i.e. GLT-1), three markers that were used to isolate progenitors, microglia, and astrocytes. **f** Percentage of cell types per donor. **g** Number of cells isolated for each cell type per plate. **h** Distribution of cells per donor and cell type. (n=3 donors; 2 females, aged 95 (504 cells) and 96 years (118 cells); 1 male, aged 72 years (116 cells)). Source data are provided as a Source Data file.

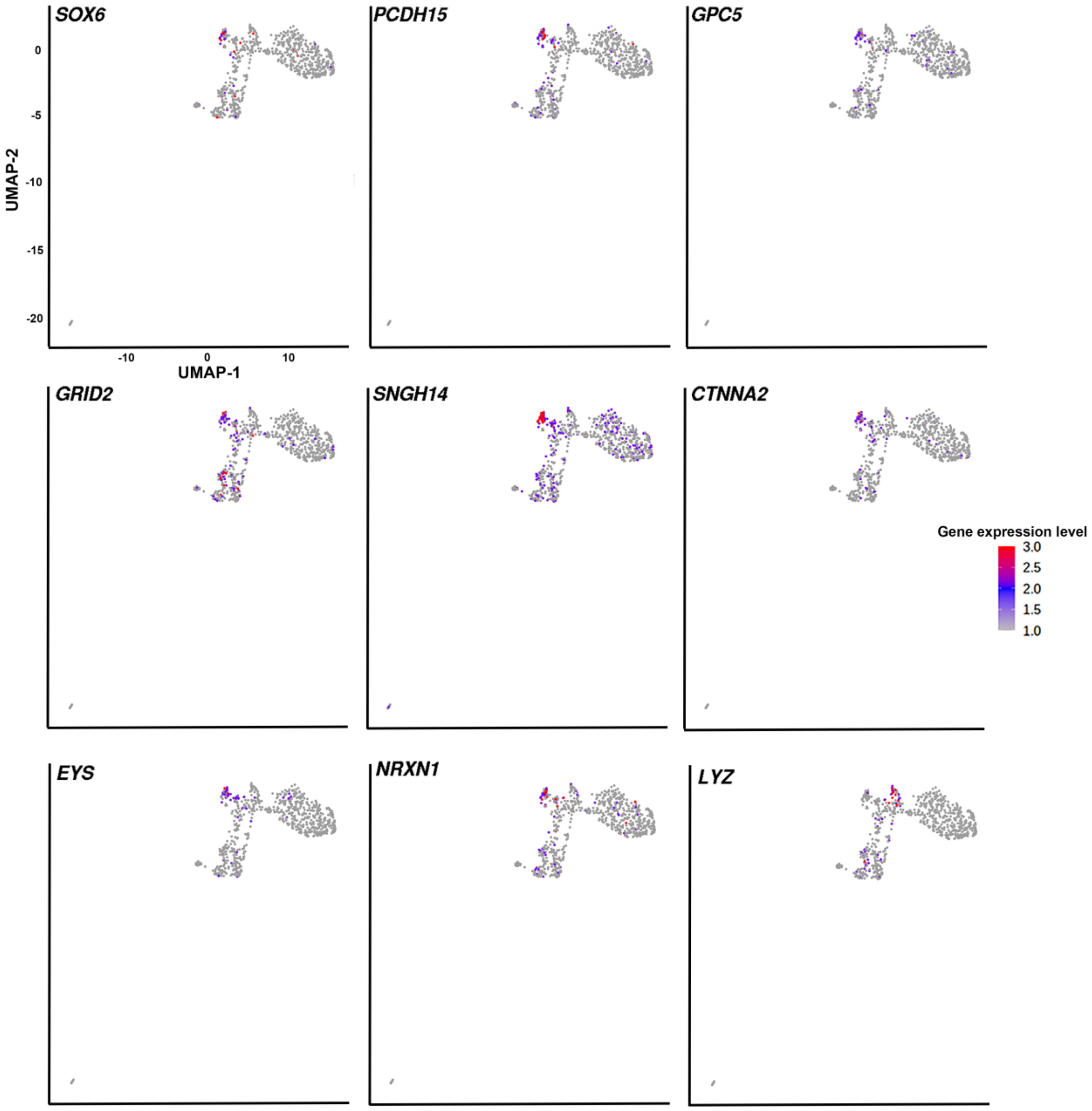

Supplementary Figure 2. **Marker genes of clusters Neuronal and X**. Feature plots showing the expression of select genes in the clusters Neuronal and X. (n=3 donors; 2 females, aged 95 (504 cells) and 96 years (118 cells); 1 male, aged 72 years (116

cells)).

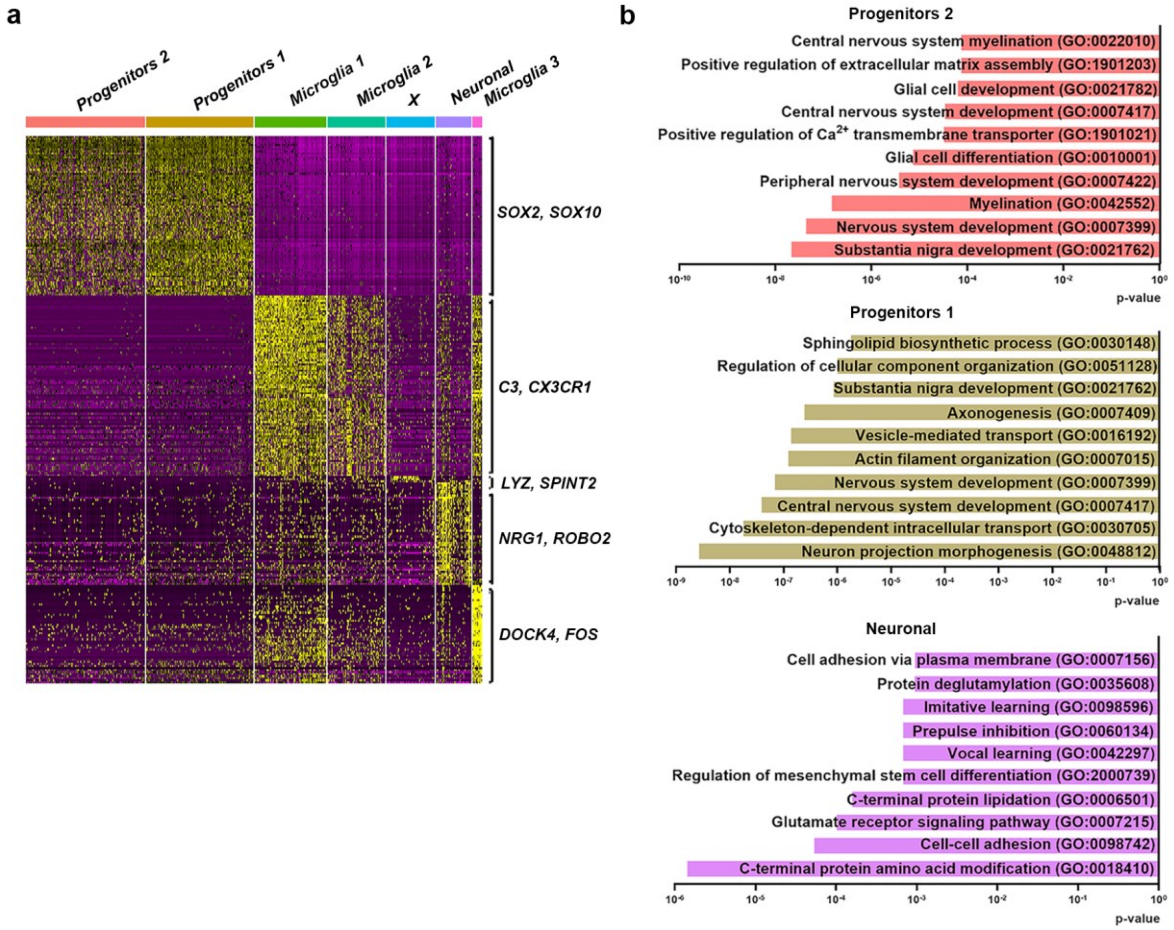

Supplementary Figure 3. **Characterization of the different cell clusters identified. a** Heatmap showing the top 50 marker genes specific to each detected cluster (yellow = high expression, purple = low expression). **b** GO analysis (biological process) with Enrichr web-based software[12,13] on cluster specific genes (only genes with an adjusted P-value < 0.01 were used to run GO analysis). P-values were calculated with the Fisher's exact test. (n=3 donors; 2 females, aged 95 (504 cells) and 96 years (118 cells); 1 male, aged 72 years (116 cells)). Source data are provided as a Source Data file.

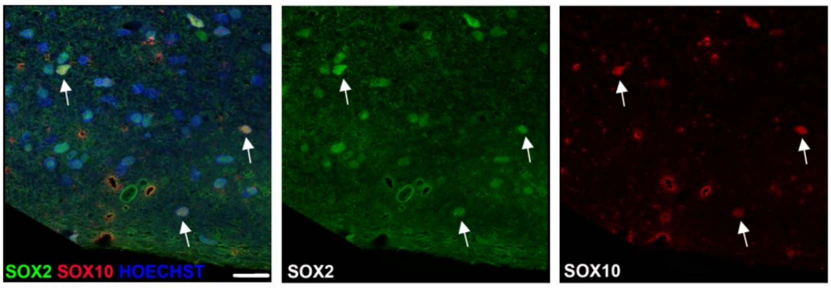

Supplementary Figure 4. **Few SOX2 positive progenitors express SOX10.** Immunofluorescence staining showing SOX2 and SOX10 expression in the SVZ of post-mortem human brain tissue (n=3 donors, 2 females aged 92 and 99 years, 1 male aged 82 years). Scale bar = 20 µm.

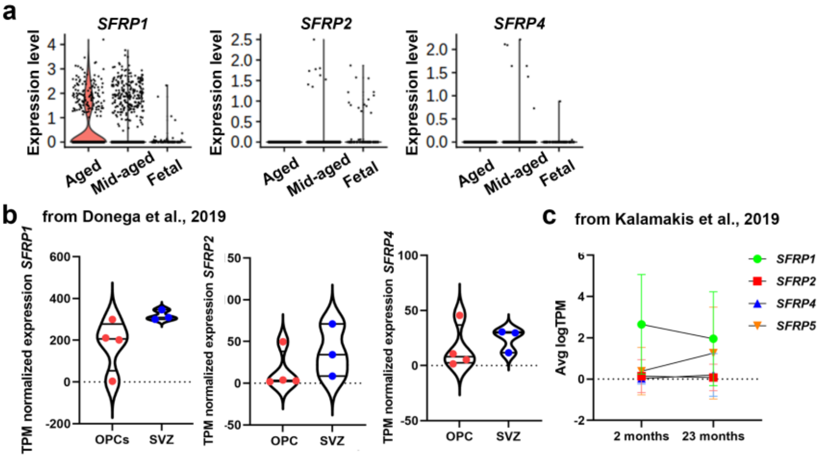

Supplementary Figure 5. **Gene expression level of different SFRP family members in NSCs. a** Violin plots showing expression level of *SFRP1*, *SFRP2* and *SFRP4*. (n=3 donors; 2 females, aged 95 (504 cells) and 96 years (118 cells); 1 male, aged 72 years (116 cells)). **b** Violin plot showing *SFRP* expression level from bulk RNA sequencing on OPCs (n=4 donors) and SVZ homogenate[1] (n=3 donors). *Sfrp* expression level in the mouse SVZ from Kalamakis et al.[20]. Source data are provided as a Source Data file.

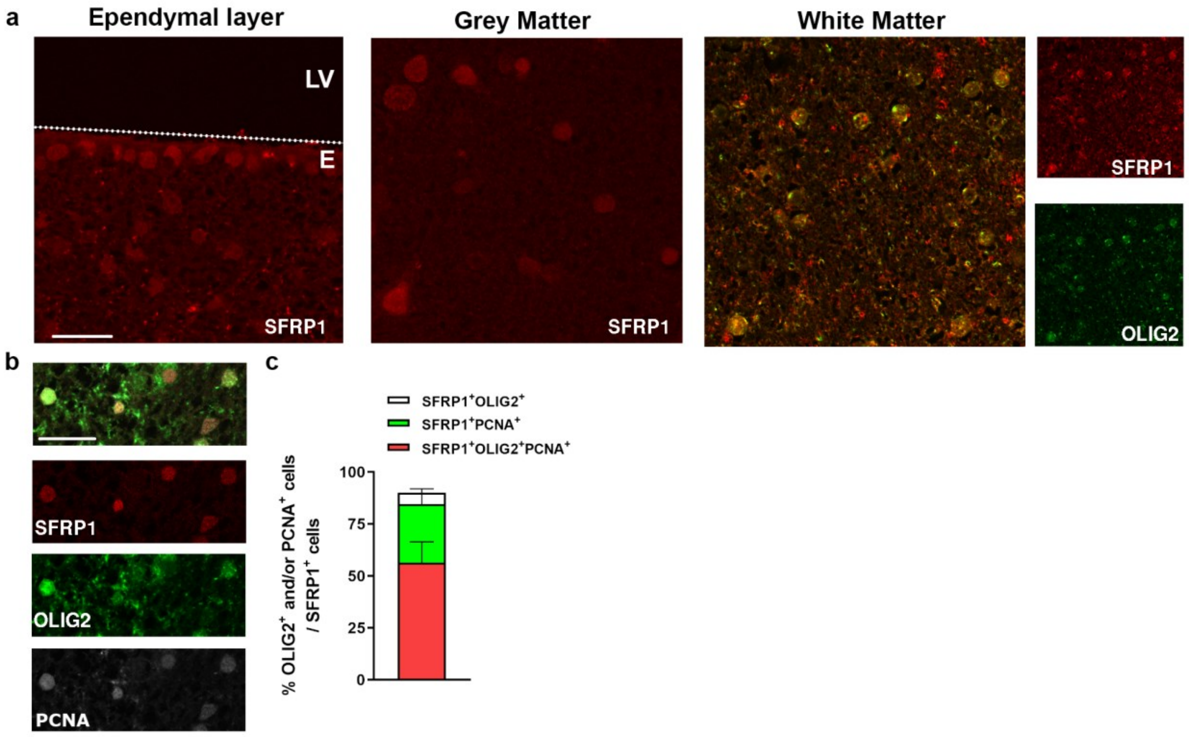

Supplementary Figure 6. **Characterization of SFRP1 expression in the adult human brain. a** SFRP1 expression outside the SVZ. SFRP1 expression can be seen in the ependymal layer (E), grey and white matter. **b** Representative image of SFRP1 co-expression of OLIG2 and PCNA in the SVZ. **c** Quantification of the proportion of SFRP1^{+} cells that express OLIG2 or PCNA (n=3 donors, 2 females, aged 92 and 99 years, 1 male aged 82 years). LV = lateral ventricle, dotted line = lateral

ventricle border. Data presented as mean ± SEM. Scale bar = 20 µm. Source data are provided as a Source Data file.

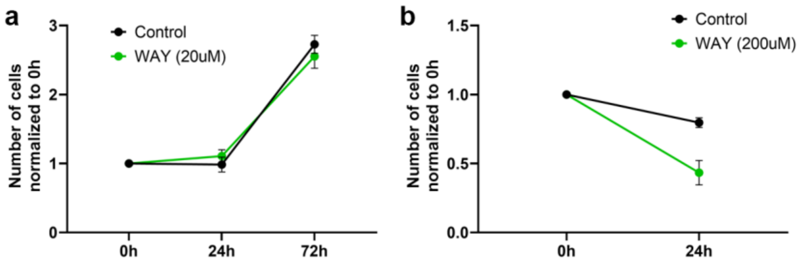

Supplementary Figure 7. **Dosage specific effect of WAY-316606.** Higher dosages of WAY-316606 of 20µM (**a**) and 200µM (**b**) did not stimulate proliferation. n=3 biological replicates. Data presented as mean ± SEM.

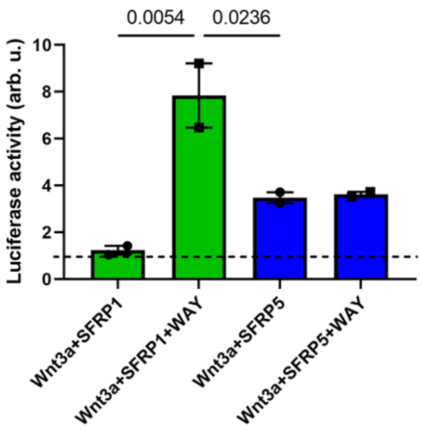

Supplementary Figure 8. **Topflash luciferase assay to determine canonical Wnt activity.** Topflash luciferase assay showing that the small molecule WAY-316606 activates the canonical Wnt pathway through inhibition of SFRP1 and not SFRP5. arb. u. = arbitrary units. Luciferase activity was corrected for the negative control (1% CHAPS, which is set at 1 arb. u.). Data presented as mean ± SEM. One-Way ANOVA with Sidák multiple comparisons test. n=2 independent experiments. Dotted line = negative control. Source data are provided as a Source Data file.

Supplementary references

1. Donega, V. *et al.* Transcriptome and proteome profiling of neural stem cells from the human subventricular zone in Parkinson's disease. *Acta Neuropathol. Commun.* **7**, 84 (2019).

2. Boutin, C., Diestel, S., Desoeuvre, A., Tiveron, M. C. & Cremer, H. Efficient in vivo electroporation of the postnatal rodent forebrain. *PLoS One* **3**, 1–6 (2008).

3. Harschnitz, O. *et al.* Autoantibody pathogenicity in a multifocal motor neuropathy induced pluripotent stem cell–derived model. *Ann. Neurol.* **80**, 71–88 (2016).

4. Falk, A. *et al.* Capture of neuroepithelial-like stem cells from pluripotent stem cells provides a versatile system for in vitro production of human neurons. *PLoS One* **7**, 1–13 (2012).

5. Koch, P., Opitz, T., Steinbeck, J. A., Ladewig, J. & Brüstle, O. A rosette-type, self-renewing human ES cell-derived neural stem cell with potential for in vitro instruction and synaptic integration. *Proc. Natl. Acad. Sci. U. S. A.* **106**, 3225–3230 (2009).

6. Chambers, S. M. *et al.* Highly efficient neural conversion of human ES and iPS cells by dual inhibition of SMAD signaling. *Nat. Biotechnol.* **27**, 275–280 (2009).

7 Hawkshaw, NJ *et al.* Identifying novel strategies for treating human hair loss disorders: Cyclosporine A suppresses the Wnt inhibitor, SFRP1, in the dermal papilla of human scalp hair follicles. *Plos Biol.* **16**, e2003705 (2018).

8. van den Brink, S. C. *et al.* Single-cell and spatial transcriptomics reveal somitogenesis in gastruloids. *Nature* **582**, (2020).

9. Dobin, A. *et al.* STAR: Ultrafast universal RNA-seq aligner. *Bioinformatics* **29**, 15–21 (2013).

10. Butler, A., Hoffman, P., Smibert, P., Papalexi, E. & Satija, R. Integrating single-cell transcriptomic data across different conditions, technologies, and species. *Nat. Biotechnol.* **36**, 411–420 (2018).

11. Hafemeister, C. & Satija, R. Normalization and variance stabilization of single-cell RNA-seq data using regularized negative binomial regression. *Genome Biol.* **20**, 296 (2019). doi:10.1101/576827

12. Chen, E. Y. *et al.* Enrichr: Interactive and collaborative HTML5 gene list enrichment analysis tool. *BMC Bioinformatics* **14**, 128 (2013).

13. Kuleshov, M. V. *et al.* Enrichr: a comprehensive gene set enrichment analysis web server 2016 update. *Nucleic Acids Res.* **44**, W90–W97 (2016).

14. Zhong, S. *et al.* A single-cell RNA-seq survey of the developmental landscape of the human prefrontal cortex. *Nature* **555**, 524–528 (2018).

15. Jäkel, S. *et al.* Altered human oligodendrocyte heterogeneity in multiple sclerosis. *Nature* **566**, 543–547 (2019).

16. Stuart, T. *et al.* Comprehensive Integration of Single-Cell Data. *Cell* **177**, 1888-1902.e21 (2019).

17. Trapnell, C. *et al.* The dynamics and regulators of cell fate decisions are revealed by pseudotemporal ordering of single cells. *Nat. Biotechnol.* **32**, 381–386 (2014).

18. Cao, J. *et al.* The single-cell transcriptional landscape of mammalian organogenesis. *Nature* **566**, 496–502 (2019).

19. Schindelin, J. *et al.* Fiji: An open-source platform for biological-image analysis. *Nat. Methods* **9**, 676–682 (2012).

20. Kalamakis, G. *et al.* Quiescence Modulates Stem Cell Maintenance and Regenerative Capacity in the Aging Brain. *Cell* **176**, 1407-1419.e14 (2019).
