# Supplementary material for: Single-cell profiling of human subventricular zone progenitors identifies SFRP1 as a target to re-activate progenitors
Source: Nat Commun. 2022 Feb 24;13:1036. doi: 10.1038/s41467-022-28626-9 (PMC8873234; doi:10.1038/s41467-022-28626-9)
Supplement: Supplementary file 2 — Description of Additional Supplementary Files [file 41467_2022_28626_MOESM2_ESM.docx]

**Description of Additional Supplementary Files:**

**Supplementary Data 1: Characteristics and pathological assessment of donors used for single-cell RNA sequencing.** Abbreviations: PMD = post-mortem delay; CSF = cerebral spinal fluid. n.d. = not determined.

**Supplementary Data 2:** **List of differentially expressed genes for each of the seven clusters.** (Bonferroni corrected two-sided Wilcoxon rank sum test). Differentially expressed genes were considered significant when adjusted P-value < 0.01.

**Supplementary Data 3: Number of cells per cluster and list of differentially expressed genes for each of the eight oligodendroglial lineage clusters.** (Bonferroni corrected two-sided Wilcoxon rank sum test). Differentially expressed genes were considered significant when adjusted P-value < 0.01.

**Supplementary Data 4:** **List of differentially expressed genes as a function of time.** Each gene was fitted in a general linear regression model with time and gene expression as variables. Significant time components were identified with coefficient ≠ 0 calculated using Wald test, and q-value was considered significant when <0.01. Multiple hypothesis testing calculated by Benjamini and Hochberg.

**Supplementary Data 5: Characteristics of control adult donors used for immunofluorescence analysis.** Abbreviations: PMD = post-mortem delay; CSF = cerebral spinal fluid.

**Supplementary Data 6: Characteristics of fetal brain tissue used for immunofluorescence analysis**. GW = gestational weeks; PMD = post-mortem delay.

**Supplementary Data 7: List of qPCR primer sequences used.**
