# Supplementary material for: Single-cell profiling of human subventricular zone progenitors identifies SFRP1 as a target to re-activate progenitors
Source: Nat Commun. 2022 Feb 24;13:1036. doi: 10.1038/s41467-022-28626-9 (PMC8873234; doi:10.1038/s41467-022-28626-9)
Supplement: Supplementary file 10 — Reporting Summary [file 41467_2022_28626_MOESM10_ESM.pdf]

nature research

Corresponding author(s): V Donega and EM Hol

Last updated by author(s): Jan 26, 2022

Reporting Summary

Nature Research wishes to improve the reproducibility of the work that we publish. This form provides structure for consistency and transparency in reporting. For further information on Nature Research policies, see our Editorial Policies and the Editorial Policy Checklist.

Statistics

For all statistical analyses, confirm that the following items are present in the figure legend, table legend, main text, or Methods section.

n/a	Confirmed	
☐	☒	The exact sample size (*n*) for each experimental group/condition, given as a discrete number and unit of measurement
☐	☒	A statement on whether measurements were taken from distinct samples or whether the same sample was measured repeatedly
☐	☒	The statistical test(s) used AND whether they are one- or two-sided<br>*Only common tests should be described solely by name; describe more complex techniques in the Methods section.*
☒	☐	A description of all covariates tested
☐	☒	A description of any assumptions or corrections, such as tests of normality and adjustment for multiple comparisons
☐	☒	A full description of the statistical parameters including central tendency (e.g. means) or other basic estimates (e.g. regression coefficient) AND variation (e.g. standard deviation) or associated estimates of uncertainty (e.g. confidence intervals)
☐	☒	For null hypothesis testing, the test statistic (e.g. *F*, *t*, *r*) with confidence intervals, effect sizes, degrees of freedom and *P* value noted<br>*Give P values as exact values whenever suitable.*
☒	☐	For Bayesian analysis, information on the choice of priors and Markov chain Monte Carlo settings
☒	☐	For hierarchical and complex designs, identification of the appropriate level for tests and full reporting of outcomes
☒	☐	Estimates of effect sizes (e.g. Cohen's *d*, Pearson's *r*), indicating how they were calculated

Our web collection on statistics for biologists contains articles on many of the points above.

Software and code

Policy information about availability of computer code

Data collection	scRNAseq analysis was performed on the Illumina NextSeq500 platform. qRT-PCR analysis was performed on Quantstudio 6 Flex (Applied Biosystems, Life Technologies). For image acquisition: software Zen black Z.1SP3 and AxioVision v4.8.2.0 were used.
Data analysis	Alignment to the human transcriptome was performed using a custom pipeline (https://github.com/anna-alemany/transcriptomics/tree/master/mapandgo/starmap). Raw reads were trimmed, aligned to the Ensembl release 75 Homo sapiens genome using RNA STAR version 2.53A (Spliced Transcripts Alignment to a Reference) and demultiplexed using cell specific barcodes. Quality check and filtering was performed on Seurat v3.2.2 using the following parameters: Only genes that were detected in at least two cells were taken for downstream analysis. Cells that had less than 100 genes or more than 3000 genes detected and that had more than 6% of their counts mapped to the mitochondrial genome were removed. Highly variable genes were identified using Seurat v3.2.2 (FindVariableFeatures function). Genes were considered marker genes when it was expressed in more than 25% of the cells with a logFC threshold of at least 0.25 (natural log). The Wilcoxon rank sum test was used to identify marker genes. P-value adjustment was performed using the Bonferroni correction. Integration of single-cell datasets published in Zhong et al., 2018 and Jäkel et al., 2019 with our dataset was done using Seurat v3.2.2 as described in Stuart et al., 2019. Assuming that there are similarities between datasets and that a subset of cells have a shared biological state, a set of molecular features could be identified. These so-called “anchors” were determined with the FindIntegrationAnchors function with dims set on 35. Genes whose expression changes over time were identified by using Monocle3 v0.2.3.0. Every gene was fitted in a linear regression model. Multiple hypothesis testing was corrected using the Benjamini and Hochberg test. Genes that had a time-dependent expression were filtered. Differentially expressed genes were considered significant when q-value < 0.01. R-Studio version 4.0.2 was used.<br><br>ImageJ v1.53c was used for quantification. An automated counting method using the Moments threshold for KI67 and SOX2, and the RenyiEntropy threshold for DAPI staining, which is provided by the software, was used to determine the number of cells positive for both markers.<br><br>qRT-PCR analysis was performed on the quantstudio Realtime PCR software (version v1.1; Applied Biosystems).

Cells were quantified using the cell counter plugin from Fiji (version 1.52p).

GraphPad Prism 7 (La Jolla, Ca, USA) was used for statistical analysis.

For manuscripts utilizing custom algorithms or software that are central to the research but not yet described in published literature, software must be made available to editors and reviewers. We strongly encourage code deposition in a community repository (e.g. GitHub). See the Nature Research guidelines for submitting code & software for further information.

Data

Policy information about availability of data

All manuscripts must include a data availability statement. This statement should provide the following information, where applicable:

- Accession codes, unique identifiers, or web links for publicly available datasets
- A list of figures that have associated raw data
- A description of any restrictions on data availability

The single-cell RNA sequencing dataset generated in this study have been deposited in NCBI's Gene Expression Omnibus database under the accession number: GSE164986 https://www.ncbi.nlm.nih.gov/geo/query/acc.cgi?acc=GSE164986). The source data generated in this study are provided in the Source Data file.

Field-specific reporting

Please select the one below that is the best fit for your research. If you are not sure, read the appropriate sections before making your selection.

☒ Life sciences ☐ Behavioural & social sciences ☐ Ecological, evolutionary & environmental sciences

For a reference copy of the document with all sections, see nature.com/documents/nr-reporting-summary-flat.pdf

Life sciences study design

All studies must disclose on these points even when the disclosure is negative.

Sample size	Because of the sparsity of fresh control brain tissue without known neurological or psychiatric disease we could not perform a sample size calculation for the work on post-mortem control brain tissue.<br>No sample size was calculated for in vitro experiments. Optimal number of cells was calculated based on pilot experiments. The number of technical replicates was also based on pilot experiments.<br>Sample size calculation was performed before carrying out the in vivo mouse experiments. Power analysis with an expected SD of 0.15 and an effect size of 0.4 yielded a number of samples of 4. For mRNA expression analysis a higher SD was used of 0.20, keeping the effect size at 0.4 yielded a number of samples of 6.
Data exclusions	Outliers were detected using the Grubbs test with $\alpha = 0.05$. Outliers were detected in data shown in Figure 7 and Figure 8. These outliers were excluded as these could be reflecting technical bias. Please see figure legends for detailed information on outliers excluded.
Replication	scRNAseq of human cells cannot be replicated in this study because of the sparsity of fresh control brain tissue without known neurological or psychiatric disease.<br>The in vitro experiments with iPSC cells had three biological replicates, and was repeated three times with different concentrations of small molecule.<br>The Luciferase assay was replicated twice.<br>Effect of WAY on proliferation was replicated in 3 different assays: in vitro on iPSCs derived NSC, in the Luciferase assay, and in vivo in mice using two different read-outs: Immunofluorescence stainings (replicated twice) and mRNA expression.
Randomization	Mouse pups from 4 different litters were randomly allocated to control group or treated group. For studies on human tissue, material was chosen based on age and disease state (that is without known neurological or psychiatric disease).
Blinding	In vitro experiments: Investigator was not blind to group conditions because of the practicality of in vitro experiments: the location of the treated and untreated wells are fixed.<br>Mouse experiments: Investigator was blind to group allocation during data allocation and analysis. Samples were coded at the beginning of the experiment and were only decoded after analysis was finished.

Reporting for specific materials, systems and methods

We require information from authors about some types of materials, experimental systems and methods used in many studies. Here, indicate whether each material, system or method listed is relevant to your study. If you are not sure if a list item applies to your research, read the appropriate section before selecting a response.

Materials & experimental systems

n/a	Involved in the study
	☒ Antibodies
	☒ Eukaryotic cell lines
☒	Palaeontology and archaeology
	☒ Animals and other organisms
	☒ Human research participants
☒	Clinical data
☒	Dual use research of concern

Methods

n/a	Involved in the study
☒	ChIP-seq
☒	Flow cytometry
☒	MRI-based neuroimaging

Antibodies

Antibodies used

For FACS: anti-GLT-1 (1:50; R&D systems, NBP1-20136); Fc-receptor (1:20; 130-059-901, Miltenyi, Bergisch Gladbach, Germany); donkey anti-rabbit AF488 (1:250; A32790, Invitrogen, Carlsbad, CA, USA); anti-CD11b-PE (1:80; 12-0118-42, Invitrogen M1/70 clone); anti-CD271 APC (1:11; 130-110-078, clone 5170131291 Miltenyi); 7-AAD (1:50; 559925, BD Pharmingen).

For Immunofluorescence staining human: rabbit anti-SOX2 (1:100; AB5603, lot Q2922255 EMDMillipore, Burlington, MA, USA); goat anti-SFRP1 (1:100; AF1384, lot IRQ061610A R&Dsystems, Minneapollis, MN, USA); mouse anti-PCNA (1:100; sc-56, Santa Cruz, Dallas, TX, USA); rabbit anti-P57 (1:100; ab75974, Abcam, Burlingame, CA, USA); rabbit anti-OLIG2 (1:100; 18953, lot 1B-327, IBL, Minneapolis, MN, USA); goat anti-SOX10 (1:200; R&Dsystems, Minneapollis, MN, USA, AF2864); Hoechst 33258 (1:5000; H3569, Invitrogen). Alexa-555 or Alexa-647 (1:1000; Invitrogen). Horseradish peroxidase antibody (1:500; Jackson ImmunoResearch, UK)

For immunofluorescence staining mouse: rabbit anti-Sox2 (1:500; AB5603, EMDMillipore), rabbit anti-Sfrp1 (1:500; ab4193, Abcam), mouse anti-Ki-67 (1:1000; ab15580, Abcam) and rabbit anti-Olig2 (1:200; IBL). Secondary antibodies conjugated to Alexa-555, Alexa-594, Alexa- 488, Alexa-647 (1:1000; Invitrogen) or Tyramide Signal Amplification (Perkin Elmer, Waltham, USA) Cy3 (1:50) or Fluorescein (1:300).

Validation

All antibodies were validated commercially. Certificates of analysis for the approved applications and relevant references are provided on manufacturer's website.

For FACS: anti-GLT-1 (https://www.novusbio.com/products/eaat2-glt1-antibody_nbp1-20136) has been tested for reactivity to human cells and flow cytometry application, anti-CD11b-PE (https://www.thermofisher.com/antibody/product/CD11b-Antibody-clone-M1-70-Monoclonal/12-0112-82) this antibody was verified by Cell treatment to ensure that the antibody binds to the antigen stated, 345 publications use this antibody for flow cytometry and it has been tested in human. Anti-CD271 APC (https://www.miltenyibiotec.com/BE-en/products/cd271-lngfr-antibody-anti-human-mouse-reafinity-rea648.html?countryRedirected=1#gref) has been tested on flow cytometry human cells.

For Immunofluorescence staining: rabbit anti-SOX2 (https://www.sigmaaldrich.com/catalog/product/mm/ab5603?lang=en&region=NL&gclid=EAIaIQobChMIlP6osInY7gIVhOJ3Ch1SyAXZEAAYAiAAEgI-dvD_BwE) this is an affinity isolated antibody purified by affinity chromatography, it recognizes the C-terminal of SOX2. It has been tested for immunofluorescence on paraffin tissue and immunocytochemistry on human cells.; goat anti-SFRP1 (https://www.rndsystems.com/products/human-sfrp-1-antibody_af1384) Detects human sFRP-1 in direct ELISAs and Western blots. In direct ELISAs, less than 2% cross-reactivity with recombinant human (rh) sFRP-2, and rhsFRP-5 is observed. Antigen affinity purified tested for immunohistochemistry and immunocytochemistry; goat anti-SOX10 (https://www.rndsystems.com/products/human-sox10-antibody_af2864) Detects human SOX10 in direct ELISAs and Western blots. Antigen affinity purified. Tested for immunohistochemistry human parffin embedded tissue; mouse anti-PCNA (https://www.scbt.com/p/pcna-antibody-pc10?gclid=EAIaIQobChMIjfi18InY7gIViuF3Ch1pwg51EAAYASAAEgJSS_D_BwE) raised against rat PCNA made in the protein A expression vector pR1T2T. Anti-PCNA Antibody (PC10) is recommended for detection of PCNA p36 protein expressed at high levels in proliferating cells of mouse, rat, human. Tested for immunohistochemistry and immunofluorescence; rabbit anti-P57 (https://www.abcam.com/p57-kip2-antibody-ep2515y-ab75974.html) Synthetic peptide corresponding to Human p57 kip2 aa 50-150 (N-terminal). RabMab technology which is a patented hybridoma based technology for making rabbit monoclonal antibodies was used. Has been tested for immunofluorescence and immunohistochemistry. abpromise guarantee.; rabbit anti-OLIG2 (https://www.ibl-america.com/olig2-anti-human-rabbit-igg-affinity-purify/) Synthetic peptide in portion of C-terminus of Human Olig2. Purified with antigen peptide. Cross-reacts with mouse and rat; rabbit anti-Sfrp1 (https://www.abcam.com/sfrp1-antibody-ab4193.html) synthetic peptide corresponding to amino acids 42 - 53 of Human SFRP1. This is an affinity purified antibody produced by immunoaffinity chromatography using the immunizing peptide after immobilization to a solid phase. Tested for immunocytochemistry and immunofluorescence; mouse anti-Ki-67 (https://www.abcam.com/ki67-antibody-ab15580.html) Immunogen affinity purified and Knock-out (KO) validation is a robust technique used to confirm antibody specificity by testing the antibody of interest in a KO cell line or tissue that does not express the target protein. Tested for mouse and human and on immunocytochemistry and immunohistochemistry. Its has over 2800 references.

Eukaryotic cell lines

Policy information about cell lines

Cell line source(s)

NSCs derived from human induced pluripotent stem cells (iPSCs) from donor OH3.1.
HEK293 cells (LGC Standards, Middlesex, UK, ATCC-CRL-11268).

Authentication	Was described previously in Harschnitz O et al., Autoantibody pathogenicity in a multifocal motor neuropathy induced pluripotent stem cell–derived model. (2016) Annals of Neurology.<br>The HEK293 cells have been authenticated by the suppliers.
Mycoplasma contamination	iPSCs and HEK293 were tested negative for mycoplasma contamination. iPSCs were frequently tested for mycoplasma infection (Lonza, LT07-318).
Commonly misidentified lines<br>(See ICLAC register)	No commonly misidentified cell lines were used in this study.

Animals and other organisms

Policy information about studies involving animals; ARRIVE guidelines recommended for reporting animal research

Laboratory animals	Animal experiments were carried out on wild-type C57BL/6j mice (Charles River, The Netherlands) aged 1 (P1), 2 (P2) or 60 (P60) days. Both males and females were used. The morning when a plug was observed is considered as E0.5 and the day of birth is defined as P0. Mice were housed in groups, with access to food and water ad libitum on a 12 hour light/dark cycle, humidity of 45-65% and a temperature between 20-24 degrees. Handling of pups was kept to a minimum to reduce stress to both mom and pups.
Wild animals	No wild animals were used.
Field-collected samples	No field collected samples were used.
Ethics oversight	All animal experiments were performed in accordance to the international guidelines from the EU directive 2012/63/EU and approved by the Experimental Animal Committee Utrecht (University Utrecht, Utrecht, Netherlands) (CCD number: AVD1150020184944).

Note that full information on the approval of the study protocol must also be provided in the manuscript.

Human research participants

Policy information about studies involving human research participants

Population characteristics	Fresh human post-mortem dorsal SVZ including adjoining white matter tissue (n=3) was obtained from donors without known neurological or psychiatric disease from the Netherlands Brain Bank (NBB; https://www.brainbank.nl), Netherlands Institute for Neuroscience, Amsterdam. Paraffin-embedded adult post-mortem dorsal SVZ tissue from donors without known neurological disease was obtained from the NBB (Supplementary Data 6) (n=5). Donors were females and males aged between 50 and 99 years. Fetal brain tissue (n=7) was obtained from males and females from gestational weeks 9, 16 and 17. For more details please see Supplementary Data 1, 5 and 6.
Recruitment	Fresh human post-mortem dorsal SVZ including adjoining white matter tissue (n=3) (Supplementary Figure 1a) was obtained from donors without known neurological or psychiatric disease from the Netherlands Brain Bank (NBB; https://www.brainbank.nl). The NBB performs quick brain autopsies to ensure high tissue quality. Directly after autopsy, samples are placed in Hibernate-A medium (ThermoFisher Scientific, Landsmeer, The Netherlands) and were kept cold until isolation. Samples had a mean post-mortem delay of 6.35 hours (Supplementary Data 1). Adult post-mortem dorsal SVZ tissue from donors without known neurological disease was obtained from the NBB (Supplementary Data 5) (n=5). Material was fixed in formalin and embedded in paraffin. All donors have given informed consent to the NBB to perform autopsies for tissue isolation and access to medical records for research purposes. To ensure donor anonymity only an autopsy serial number, which is given by the NBB, is disclosed. This number contains the year that the autopsy was performed and the number of the autopsy. Fetal human brain tissue was obtained from abortion material without developmental structural chromosomal abnormalities (Supplementary Data 6) (n=7) from the Chinese University of Hong Kong.
Ethics oversight	Foetal brain tissue:<br>Donors of fetal tissue have given informed consent to the use of the tissue for research. Women being admitted for pregnancy termination due to different clinical indications were invited to donate their conception material, including placenta, abortus and fetal blood for study. The participants understood that the fetal tissue would normally be discarded as medical waste. Participation was entirely voluntary, without compensation, and the decision of the donor did not interfere with the clinical management. Fetal tissue samples without structural and chromosomal abnormalities, were collected after the completion of the termination of the pregnancy. Involvement in the study did not confer any additional risk over the routine clinical treatment. The participants had the right to withdraw from the study and request for the collected samples to be destroyed at any moment. All clinical information will remain confidential. Identity of the donor is kept anonymous by the use of a serial number. This study was performed according to the Dutch, European, and Hong Kong institutional ethical regulations for the use of human and abortion material. This study was approved by the Chinese University of Hong Kong – New Territories East Cluster Clinical Research Ethics Committee (CREC), Faculty of Medicine, The Chinese University of Hong Kong (under the human ethics approval reference number CREC-2004.330).<br>The study design and conduct complied with all relevant regulations regarding the use of human study participants and was conducted in accordance with the criteria set by the Declaration of Helsinki.<br>Adult brain tissue:<br>Freshly isolated tissue:<br>All donors have given informed consent to the NBB to perform autopsies for tissue isolation, access to medical records for research purposes and consent to publish clinical information potentially identifying individuals. The independent Medical Ethics Committee review board of the VU University Medical Center has reviewed and agreed to the procedures of the NBB concerning donation of brain material for scientific research (2009/148) (https://www.brainbank.nl/about-us/the-nbb/).<br>Autopsies are performed by the NBB at the designated premises of the VU Medical Center in Amsterdam, the Netherlands.

The NBB adheres to the standards for quality, safety and ethics for obtaining and handling of human tissue, as described in BrainNet Europe's Code of Conduct for brain banking. This study was reviewed and authorized by NBB’s scientific committee. The study design and conduct complied with all relevant regulations regarding the use of human study participants and was conducted in accordance with the criteria set by the Declaration of Helsinki. This study was performed according to the Dutch and European legal and ethical regulations. To ensure donor anonymity only an autopsy serial number, which is given by the NBB, is disclosed.

Paraffin embedded tissue:

All donors have given informed consent to the NBB to perform autopsies for tissue isolation, access to medical records for research purposes and consent to publish clinical information potentially identifying individuals. The independent Medical Ethics Committee review board of the VU University Medical Center has reviewed and agreed to the procedures of the NBB concerning donation of brain material for scientific research (2009/148) (https://www.brainbank.nl/about-us/the-nbb/). Autopsies are performed by the NBB at the designated premises of the VU Medical Center in Amsterdam, the Netherlands.The NBB adheres to the standards for quality, safety and ethics for obtaining and handling of human tissue, as described in BrainNet Europe's Code of Conduct for brain banking. This study was reviewed and authorized by NBB’s scientific committee. The study design and conduct complied with all relevant regulations regarding the use of human study participants and was conducted in accordance with the criteria set by the Declaration of Helsinki. This study was performed according to the Dutch and European legal and ethical regulations. To ensure donor anonymity only an autopsy serial number, which is given by the NBB, is disclosed..

Note that full information on the approval of the study protocol must also be provided in the manuscript.
